# Traditional Herbal Medicines Against CNS Disorders from Bangladesh

**DOI:** 10.1007/s13659-020-00269-7

**Published:** 2020-10-14

**Authors:** Md. Josim Uddin, Christian Zidorn

**Affiliations:** 1 Pharmazeutisches Institut, Abteilung Pharmazeutische Biologie, Christian-Albrechts-Universität zu Kiel, Gutenbergstraße 76, 24118 Kiel, Germany; 2 Department of Pharmacy, Faculty of Science and Engineering, International Islamic University Chittagong, Chittagong, 4318 Bangladesh

**Keywords:** CNS disorder, Medicinal plants, Traditional plants, Ethnopharmacology, Review

## Abstract

**Abstract:**

The majority of the population in Bangladesh uses traditional plant-based medicines to manage various ailments, including central nervous system (CNS) disorders. This review presents ethnobotanical information and relevant scientific studies on plants used in traditional healthcare for the management of various CNS disorders in Bangladesh. The information on the medicinal plants of Bangladesh effective against CNS disorders published in scientific journals, books, and reports was compiled from different electronic databases using specific key words. The present article provides comprehensive information on a total of 224 medicinal plant species belonging to 81 families used for the treatment of CNS disorders by the various peoples of Bangladesh. In total, we reviewed more than 290 relevant papers. In this study, leaves were found as the most often used plant organ, followed by roots, fruits, whole plants, barks, seeds, stems, rhizomes, and flowers. The Fabaceae family contributes the highest number of used species, followed by Rubiaceae, Lamiaceae, Cucurbitaceae, Vitaceae, Euphorbiaceae, Malvaceae, and Zingiberaceae. The most frequently used species (in decreasing order) are *Asparagus racemosus, Centella asiatica, Stephania japonica*, *Aegle marmelos, Coccinia grandis, Tabernaemontana divaricata*, *Bacopa monnieri*, *Abroma augusta,* and *Scoparia dulcis*. This review may serve as a starting point for a rational search for neuroactive natural products against CNS disorders within the Flora of Bangladesh.

**Graphic Abstract:**

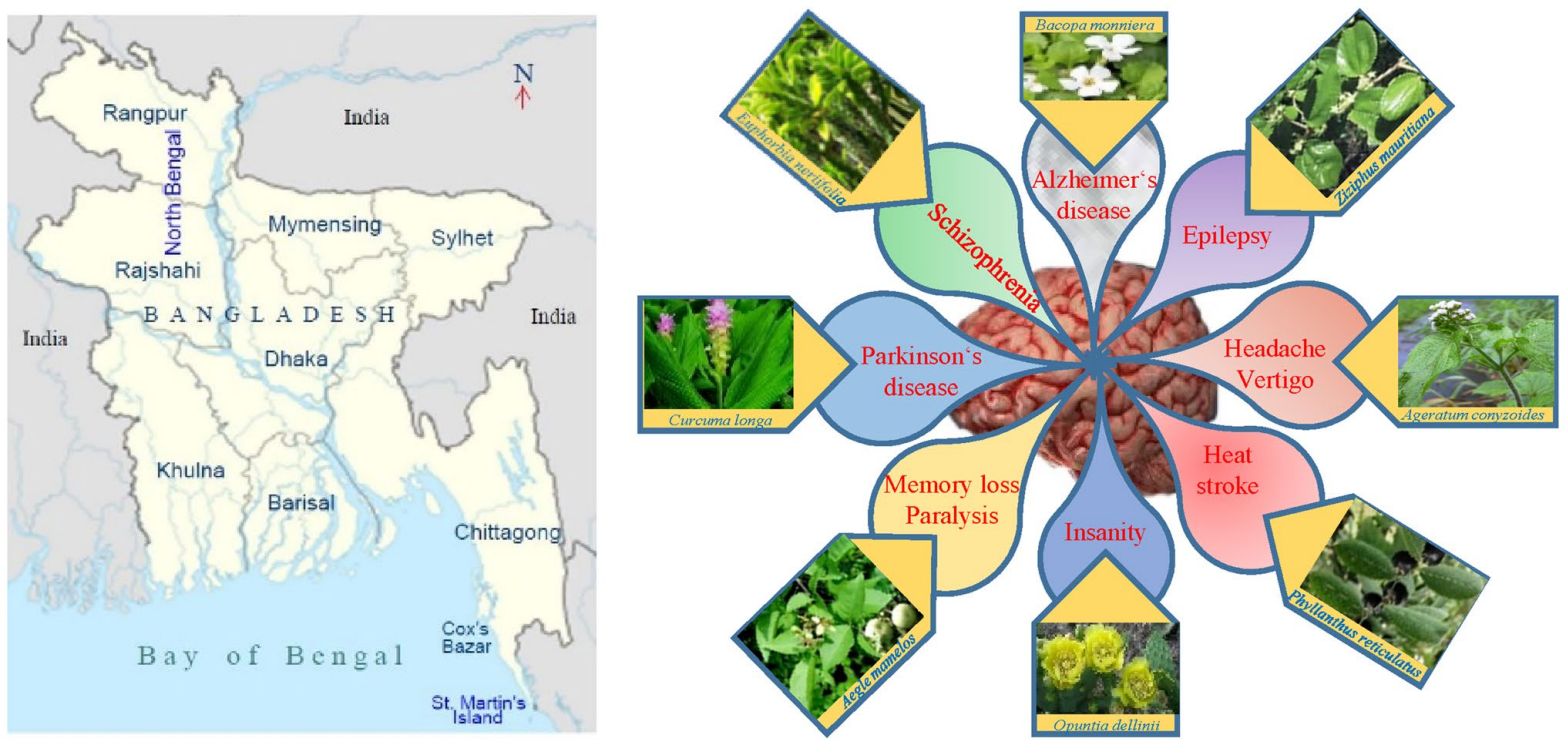

## Introduction

The central nervous system (CNS), as an integral part of the nervous system, is associated with a number of important functions and mainly consists of the brain and the spinal cord. A CNS disorder refers to a disease that affects the structure or function of brain (encephalopathy) or spinal cord (myelopathy) causing neurological or psychiatric or neurodegenerative complications. Neuroprotection denotes strategies to defend the central nervous system (CNS) against a number of factors such as structural defects, infections, neuronal injury, autoimmune disorders, tumors, neurodegeneration, and others, which may lead to CNS disorders [1]. In recent years, these disorders are rising due to the increase of life expectancy, and thus place a tremendous burden on families and social economies. A new report from the World Health Organization (WHO) shows that neurological disorders affect up to one billion people worldwide, among them 6.8 million people die every year. In addition, the prevalence of CNS disorders is around two times higher in developing countries than in the developed world [2].

Herbal supplements have long played important roles to treat various neuronal and pathological disorders without or with limited side effects. During recent years, complementary and alternative medicine (CAM) has become more popular worldwide. Many plant species have emerged as herbal medicines, and their active components have been subjected to extensive scientific research around the world [3–5]. CAM or traditional medicines are considered safe and effective in sensitive and complicated diseases like CNS disorder, while having less side effects than synthetic compounds [6]. Newman and Cragg reported that more than two thirds of the active agents recently introduced into the market have some relationship to natural sources and only 30% of new chemical entities used as medicines are of purely synthetic origin [7]. The knowledge of ethnobotany therefore continues to provide a valuable starting point for many successful drug-screening projects [8].

Also in western societies, there has been an increasing interest in herbal medicines, which are often perceived as more ‘natural’ and ‘softer’ treatments compared to synthetic drugs [9]. Drug discovery based on traditional knowledge has been termed ‘reverse pharmacology’; in this approach, drug candidates are first identified based on large-scale usage in the population before initiating clinical trials. This approach can cut the time span, needed for drug discovery, from on average twelve years (classical approach) to five years or even less (reverse pharmacology); the latter has the additional advantage of far lower development costs [10].

Traditional knowledge of medicinal plants as a complementary and alternative therapy has additionally the great significance for conserving cultural traditions and identities. Moreover, community healthcare is fostered and interesting leads for future drug development projects can be found. From this perspective, ethnopharmacological data of medicinal plants on CNS disorders will ease the identification of important species utilized in traditional medicine**.** In this review, we summarize ethnopharmacological knowledge of all currently known popular CNS active herbal remedies in Bangladesh. Additionally, we provided more details on six selected species: *Bacopa monnieri*, *Centella asiatica, Curcuma longa*, *Cyperus rotundus*, *Morinda citrifolia*, and *Withania somnifera* (author citations for these and all other scientific species names mentioned in this text have been consistently omitted from the main body of the text, but are provided in Table 1). This review on species from Bangladesh is intended to stimulate the interest in a deeper evaluation of the mentioned species as potential sources for structurally and functionally novel CNS active drug leads or hits.

**Table 1:** Plant species along with their experimental records used for care of CNS disorders in Bangladesh

Families	Plant species	Local name	Life-form	Used part	Types of CNS disorder	Experimental evidence on CNS disorder	References
Acanthaceae	*Andrographis paniculata* (Burm.f.) Wall.	Kalomegh	Herb	Leaves	Vertigo	Increase cognitive functions	[86, 87]
	*Justicia gendarussa* Burm.f.	Nilnishinda	Undershrub	Leaves	Paralysis	NRE	[88]
	*Staurogyne argentea* Wall.	Ranga Jari (tribal)	Herb	Leaves	Mental disorder	NRE	[89]
Acoraceae	*Acorus calamus* L.	Bach	Herb	Rhizome, leaves	Paralysis, epilepsy, heat stroke (Kh)	Increase cognitive function	[90–92]
Amaranthaceae	*Achyranthes aspera* L.	Apang	Herb	Whole plant	Epilepsy, paralysis	Attenuate epilepsy, Anticonvulsant	[93–96]
	*Aerva lanata* (L.) Juss.	Chaya	Herb	Whole plant	Headache	NRE	[97]
	*Amaranthus viridis* L.	Notey shak	Herb	Leaves	Epilepsy	NRE	[98]
	*Cyathula prostrata* Blume	Uphutlengra	Forb/herb	Leaves, root	Epilepsy, headache (Ma)	Antinociceptive	[89, 99, 100]
Anacardiaceae	*Magnifera sylvatica* Roxb.	Jongli aam	Tree	Young shoot	Headache	NRE	[101]
	*Semecarpus anacardium* L.f.	Bhela	Tree	Fruit	Nervous debility	Neuroprotective	[102, 103]
Apiaceae	*Centella asiatica* (L.) Urb.	Thankuni	Herb	Leaves	Memory loss, mental disorder, insanity	Alzheimer's disease, Parkinson’s disease	[14, 47, 89, 104–106]
	*Foeniculum vulgare* Mill	Mouri	Herb	Fruit, seed	Nervous debility, headache	Enhances cognitive function and memory	[107–109]
Apocynaceae	*Alstonia scholaris* (L.) R.Br.	Satim	Tree	Bark	Nervous debility (Or)	anti-dopaminergic (schizophrenia)	[88, 110, 111]
	*Calotropis gigantea* (L.) W.T.Aiton	Bara akand	Shrub	Shoot	Paralysis (Or)	Alzheimer's disease and Parkinson's disease	[91, 111, 112]
	*Carissa carandas* L.	Karamcha	Tree	Fruit	Insanity, headache	Anticonvulsant	[113–115]
	*Hemidesmus indicus* (L.) R.Br.	Anantamul	Shrub	Leaves, root	Mental disorder, nervous debility, heat stroke	Anticholinesterase activity	[113, 114, 116]
	*Marsdenia tenacissima* Moon	Chitti	Herb	Leaves	Paralysis	NRE	[89]
	*Rauvolfia serpentina* Benth.	Sarpagandha	Undershrub	Leaves, root	Epilepsy, insanity, vertigo, schizophrenia	Acetylcholinesterase inhibition	[13, 89, 117]
	*Tabernaemontana divaricata* (L.) R.Br.	Tagar	Shrub	Leaves, root, flower	Paralysis, epilepsy	Alzheimers’s disease	[118–122]
	*Thevetia peruviana* (Pers.) K.Schum.	Holde, Korobi	Shrub	Bark, seed	Schizophrenia	Anti-acetylcholinesterase	[4, 123]
Araceae	*Alocasia macrorrhizos* (L.) G.Don	Mankachu	forb/herb	Petiole	Mental disorder	NRE	[124]
	*Colocasia esculenta* (L.) Schott	Mukhikachu	Forb/herb	Whole plant	Nervous system disorder	Nerve tonic	[14, 125]
	*Pothos scandens* L.	Sunat	Epiphyte	Leaves	Migraine, vertigo	NRE	[101]
	*Typhonium trilobatum* (L.) Schott	Ghetkaachu	Herb	Whole plant	Nervous debility, mental disorder	NRE	[89, 126]
	*Xanthosoma violaceum* Schott	Shada kochu	Forb/herb	Tuber	Alzheimer’s disease (Ga)	Antinociceptive	[127, 128]
Araliaceae	*Schefflera roxburghii* Gamble	Den anno	Tree	Leaves	Insomnia	NRE	[129]
	*Trevesia palmata* Vis.	Argoza	Shrub	Leaves, fruit, root	Paralysis	NRE	[129]
Arecaceae	*Areca catechu* L.	Shupari	Palm	Fruit	Heat stroke (Sa)	Alzheimer's disease, Antidepressant	[130–132]
	*Borassus flabellifer* L.	Tal	Palm	Fruit	Epilepsy	NRE	[88]
	*Phoenix sylvestris* (L.) Roxb.	Khejur	Palm	Root, Fruit	Nervous debility	CNS depressant	[14, 94, 133]
Asclepiadaceae	*Hoya parasitica* Wall.	Cherapata	Epiphyte	Leaves	Paralysis	NRE	[101]
Asparagaceae	*Asparagus racemosus* Willd.	Sotamuli	Undershrub	Root	Mental disorder (Be), nervous debility, memory loss, epilepsy	Improves cognition, enhances memory, amnesia	[14, 88, 91, 134, 135]
	*Dracaena spicata* Roxb.	Dracaena	Shrub	Leaves	Paralysis	NRE	[114]
Asteraceae	*Cyanthillium patulum* H.Rob.	Kukurshunga	Herb	Leaves, root, flower	Vertigo	NRE	[101, 136]
	*Eclipta prostrata* L.	Kesuti	Herb	Leaves, root	Brain tonic, vertigo	Nootropic and anxiolytic activity	[101, 137]
	*Enhydra fluctuans* Lour.	Helencha	Herb	Whole plant	Nervous system disorder	CNS depressant	[126, 138]
	*Synedrella nodiflora* Gaertn.	Relanodi	Herb	Leaves, stem	Vertigo	Antipsychotic properties	[101, 139]
Athyriaceae	*Diplazium esculentum* (Retz.) Sw	Dhekishak	Fern	Leaves	Headache, epilepsy, paralysis	Cholinesterase and NADH oxidase inhibition	[106, 118, 140]
Begoniaceae	*Begonia silhetensis* (A.DC.) C.B.Clarke	Goni kanti	Herb	Leaves	Headache	NRE	[89]
Bignoniaceae	*Campsis radicans* (L.) Seem	Egro (Ma)	Vine	Leaves	Headache (Ma)	NRE	[99]
	*Crescentia cujete* L.	Jummu makal	Tree	Bark, fruit	Brain disorder (Mental disorder)	CNS depressant	[141, 142]
	*Oroxylum indicum* (L.) Benth.	Khona	Tree	Stem	Mental disorder (Be)	Parkinson’s disease, neurogenin 2 promoter activator	[91, 143, 144]
Bixaceae	*Bixa orellana* L.	Latkan	Tree	Seed	Epilepsy	Reduce oxidative stress in brain	[91, 145]
Boraginaceae	*Heliotropium indicum* L.	Hatishura	Forb/herb	Leaves	Heat stroke, mental disorder	NRE	[113, 146]
	*Tournefortia roxburghii* C.B.Clarke	Shamshog	Climber	Leaves	Mental disorder, paralysis	NRE	[89]
Brassicaceae	*Brassica napus* L.	Sarisha	Herb	seed	Mental disorder (Be)	NRE	[91]
Burseraceae	*Canarium euphyllum* Kurz	Sheti dhup	Tree	Bark	Headache, insomnia	NRE	[146]
Cactaceae	*Cereus grandiflorus* (L.) Mill.	Kuth-raaz	Herb	Whole plant	Nervous system disorder	NRE	[147]
	*Opuntia dillenii* Haw.	Phanimansa	Shrub	Leaves	Paralysis (Tr), insanity, headache	Neurodegenerative disease	[141, 148, 149]
Cannabaceae	*Cannabis sativa* L.	Bhang, Siddhi	Herb	Leaves	Schizophrenia	Neurodegenerative diseases, Alzheimer’s disease	[4, 150, 151]
	*Trema orientalis* (L.) Blume	Jibon	Tree	Whole plant	Nervous debility	Anticholinesterase activity	[108, 152]
Capparaceae	*Crateva religiosa* G. Forst	Barun	Tree	Bark, leaves	Mental disorder (Be)	Glutamatergic neurotransmission	[91, 153]
Cleomaceae	*Cleome diffusa* Roxb.	Sultae	Forb/herb	Leaves		NRE	[141]
Combretaceae	*Terminalia arjuna* (Roxb.) Wight & Arn.	Arjun	Tree	Bark	Nervous debility, paralysis	Protects neurons from cerebral ischemia	[88, 154]
	*Terminalia bellirica* (Gaertn.) Roxb.	Bahera	Tree	Fruit	Paralysis, headache	Tranquilizer	[14, 114, 155]
	*Terminalia chebula* Retz	Haritaki	Tree	Fruit	Brain disorder (mental disorder)	Protects ischemic neuronal damage	[88, 107, 156]
Commelinaceae	*Amischotolype mollissima* Hassk.	Molisima	Herb	Root	Epilepsy	NRE	[101]
Compositae	*Ageratum conyzoides* L.	Dochunti	Herb	Whole plant	Headache, Paralysis (Kh) Vertigo	Antinociceptive	[89, 99, 118, 157]
	*Blumea balsamifera* DC.	Kakronda	Shrub	Leaves	Headache, insomnia	Inhibition of NO (Alzheimer's disease)	[158, 159]
	*Emilia sonchifolia* DC.	Sadimodi	Herb	Leaves	Paralysis (Ch)	Antinociceptive	[160, 161]
	*Eupatorium ayapana* Vent.	Ayapan	Herb	Leaves	Epilepsy	Sedative, anxiolytic, and antidepressive	[89, 162]
	*Gynura nepalensis* DC.	Dhup baisak (Ch)	Herb	Leaves	Paralysis (Ch)	NRE	[160]
Convolvulacea	*Convolvulus pluricaulis* Choisy	Shonkapuspo	Herb	Leaves, flower	Neurosis, epilepsy	Neuroprotective	[86, 163]
	*Ipomoea aquatica* Forssk.	Kalmi Shak	Vine	Whole plant	Nervous system disorder, headache	CNS depressant, memory and Alzheimer’s disease	[126, 164–166]
	*Ipomoea mauritiana* Jack	Bhui kumra	Vine	Leaves, root	Headache, insomnia	NRE	[146]
Costaceae	*Cheilocostus speciosus* C.D.Specht *(Costus speciosus* Sm.)	Banduki	Forb/herb	Whole plant	Mental disorder (Be), paralysis	Neuroinflammatory diseases	[89, 91, 167]
Crassulaceae	*Bryophyllum pinnatum* Kurz	Pathorkuchi	Herb	Leaves	Epilepsy, headache (Ma), vertigo	Neurosedative	[99, 168]
	*Kalanchoe pinnata* (Lam.) Pers.	Patharkuchi	Subshrub	Leaves	Epilepsy, headache	CNS depressant	[14, 141, 147, 169]
	*Kalanchoe spathulata* DC.	Himsagor	Subshrub	Leaves	Headache	NRE	[141]
Cucurbitaceae	*Benincasa hispida* (Thunb.) Cogn.	Chalkumra	Vine	Seed, fruit	Epilepsy, nervous system disorder	Management of depressive illness	[170]
	*Citrullus lanatus* (Thunb.) Mansf.	Tarmuj	Climber	Fruit, seed	Brain tonic (Nervous debility)	Neurodegenerative diseases	[171, 172]
	*Coccinia grandis* (L.) Voigt	Telakucha	Climber	Leaves	Mental disorder, Paralysis, Schizophrenia, heat stroke, headache	Chemoprotective in brain	[4, 14, 98, 108, 173, 174]
	*Cucumis callosus* Cogn.	Bangi	Vine	Fruit, seed	Memory loss, vertigo	NRE	[171]
	*Lagenaria vulgaris* Ser.	Lau, Kadu	Vine	Fruit	Heat stroke, Headache	NRE	[113, 171]
	*Solena amplexicaulis* (Lam.) Gandhi	Kundri	Shrub	Leaves	Epilepsy, mental disorder	NRE	[89]
	*Trichosanthes bracteata* (Lam.) Voigt	Makalphal	Climber	Fruit, seed	Headache	NRE	[97]
	*Trichosanthes cucumerina* L.	Chichinga	Climber	Fruit, seed	Headache	NRE	[129]
Cyperaceae	*Cyperus rotundus* L.	Mutha, Takudare (Sa)	Graminoid	Root	Paralysis, (Sa)	Modulate memory impairment	[175, 176]
Dilleniaceae	*Dillenia indica* L.	Chalta	Tree	Fruit	Epilepsy, headache	Inhibit diabetic neuropathic pain	[114, 177, 178]
Dioscoreaceae	*Dioscorea bulbifera* L.	Banalu	Climber	Aerial part, tuber	Headache	NRE	[101]
	*Dioscorea pentaphylla* L.	Thubri	Vine	Leaves	Paralysis	NRE	[114]
Droseraceae	*Drosera indica* L.	Mukhjali	Herb	Whole plant	Headache	NRE	[179]
Euphorbiaceae	*Acalypha indica* L.	Muktajhuri	Herb	Whole plant	Insanity	NRE	[107]
	*Croton caudatus* Geiseler	Sabarjala	Shrub	Root, leaves	Paralysis (Ch)	NRE	[160]
	*Euphorbia neriifolia* L.	Monshaseez	Tree	Leaves	Schizophrenia	Anti-anxiety, anti-psychotic, anti-convulsant	[4, 180]
	*Euphorbia tirucalli* L.	Lanka Sij	Tree	Stem	Paralysis (Ba)	CNS depressant	[181, 182]
	*Macaranga denticulate* Müll.Arg.	Dati bura	Tree	Leaves, flower	Epilepsy	NRE	[101]
	*Macaranga peltata* Müll.Arg.	Pelta bura	Tree	Bark, root	Paralysis	NRE	[101]
	*Pedilanthus tithymaloides* (L.) Poit.	Barakut (Ch)	Shrub	whole plant	Headache (Ch)	Sedative	[160, 183]
Fabaceae	*Cassia occidentalis* L.	Kalkasunde	Undershrub	Leaves, fruit	Paralysis	NRE	[14]
	*Desmodium gangeticum* DC.	Alpani	Shrub	Leaves, root	Mental disorder	CNS depressant	[101]
	*Desmodium triquetrum* DC.	Komorsina	Undershrub	Root	Epilepsy, paralysis (Ch)	NRE	[89, 129, 184]
	*Mucuna pruriens* DC.	Alkushi	Climber	Root, seed	Nervine tonic	Neuroprotection for Parkinson's disease	[101, 185]
	*Saraca indica* L.	Ashok	Tree	Leaves, bark	Nervous debility	Antidepressant	[186, 187]
Lamiaceae	*Callicarpa arborea* Roxb.	Bormala	Tree	Leaves, bark, root, stem	Epilepsy	NRE	[101]
	*Clerodendrum indicum* Kuntze	Bamunhatti	Shrub	Leaves, root	Epilepsy	NRE	[99]
	*Clerodendrum viscosum* Vent.	Bhat	Shrub	Leaves	Paralysis	CNS depressant	[89, 188]
	*Leucas aspera* Link	Donkolos	Herb	Whole plant	Headache (Kh, Ma)	NRE	[99, 118]
	*Leucas zeylanica* (L.) R.Br.	Kusha	Herb	Leaves, flower	Epilepsy, headache (Kh), insomnia (Kh)	NRE	[99]
	*Ocimum americanum* L.	Radha tulshi	Undershrub	Leaves, seed	Schizophrenia	Anti-cholinesterase activity	[4, 189]
	*Ocimum gratissimum* L.	Ram Tulsi	Subshrub	Whole plant	Paralysis, mental disorder (Be), headache	Neurodegenerative disorder	[91, 119, 160, 190]
	*Premna corymbosa* Merr.	Ganiari	Shrub	Root	Neurological problem	Antinociceptive	[179, 191]
	*Vitex negundo* L.	Nishinda	Small tree	Leaves	Schizophrenia, headache	Reduce cerebral oxidative stress	[4, 192–194]
	*Vitex peduncularis* Wall.	Horina	Tree	Leaves, bark, root	Epilepsy	NRE	[101]
Lauraceae	*Actinodaphne obovate* Blume	Kula pata	Tree	Leaves, root	Epilepsy, mental disorder	NRE	[101]
	*Litsea polyantha* Juss.	Uruijja, Menda	Tree	Bark	Schizophrenia	CNS depressant, anti-convulsant	[4, 195, 196]
Lecythidaceae	*Barringtonia acutangula* (L.) Gaertn.	Hijal	Tree	Seed, root bark	Headache	CNS depressant activities	[86, 170]
Leguminosae	*Abrus precatorius* L.	Kuch	Climber	Root, seed	Headache, Paralysis	Neuroinflammatory disorder	[97, 179, 197]
	*Acacia farnesiana* (L.) Willd.	Belatibabla	Tree	Flower, leaves, root	Vertigo, headache (Ch)	Antinociceptive	[99, 129, 198]
	*Adenanthera pavonina* L.	Rokto chondon	Tree	Wood	Headache (Sa)	NRE	[199]
	*Bauhinia acuminata* L.	Kanchan	Tree	Root, flower	Mental disorder, epilepsy	NRE	[89]
	*Caesalpinia crista* L.	Baghinjanum (Sa)	Climber	Fruit, seed	Headache (Sa)	Alzheimer's disease	[89, 200]
	*Cassia fistula* L.	Sonalu	Tree	Leaves, fruit, root	Epilepsy, nervous debility	Antinociceptive	[14, 113, 201]
	*Clitoria ternatea* L.	Aparajita	Herb	Flower	Memory loss	Enhances cognitive function	[202–204]
	*Codariocalyx motorius* H.Ohashi, (*Desmodium motorium* Merr.)	Gorachand	Shrub	Leaves	Mental disorder (Be)	NRE	[91]
	*Crotalaria pallida* Aiton	Jhun Jhuni	Undershrub	Whole plant	Paralysis	Central inflammatory diseases	[2, 205]
	*Erythrina variegate* L.	Mandar	Tree	Leaves, seed	Epilepsy	NRE	[86]
	*Mimosa diplotricha* C.Wright	Bra lojjaboti	Shrub	Seed, root	Mental disorder (Be)	NRE	[91]
	*Mimosa pudica* L.	Lajjaboti	Undershrub	Whole plant	Insomnia	Memory enhance, 5-HT neuronal activity	[113, 206, 207]
	*Senna tora* Roxb.	Chakunda	Forb/herb	Leaves	Mental disorder, insanity	Alzheimer’s disease, amyloid-beta induced diseases	[89, 208, 209]
	*Sesbania cannabina* (Retz.) Poir.	Lal chainche	Shrub	Root, bark, leaves	Epilepsy	NRE	[94]
	*Sesbania grandiflora* Poir.	Bock phool	Tree	Leaves	Epilepsy (Sa)	Neuroprotective	[199, 210]
	*Uraria crinita* (L.) DC.	Diangleja	Shrub	Whole plant	Paralysis (Ch)	NRE	[184]
	*Uraria prunellaefolia* Graham	Bilai-langur	Undershrub	Root	Epilepsy (Ch)	NRE	[129]
Lygodiaceae	*Lygodium flexuosum* (L.) Sw.	Shona jhuri	Climber	Leaves, stem, root	Headache, mental disorder, epilepsy	NRE	[101, 141]
	*Lygodium altum* Alderw.	Dheki Shak	Fern	Whole plant	Epilepsy, mental disorder, headache (Kh, Tr)	NRE	[99]
Lythraceae	*Lawsonia inermis* L.	Mehedi	Shrub	Leaves	Mental disorder (Be), epilepsy	Enhances memory	[91, 93, 211]
Malvaceae	*Abroma augusta* (L.) L.f.	Ulothkombal	Shrub	Fruit, flower	Schizophrenia, Heat stroke, mental disorder (Ta)	CNS depressant	[4, 113, 212, 213]
	*Grewia laevigata* Vahl	Monsimais (Ch)	Herb	Leaves, root, bark	Paralysis (Ch)	NRE	[160]
	*Grewia serrulata* DC.	Panicherra	Tree	Leaves, root	Paralysis	NRE	[101]
	*Pterospermum acerifolium* (L.) Willd.	Kanokchapa	Tree	Flower	Brain disorder (Mental disorder) (Sa)	NRE	[176]
	*Sida acuta* Burm.f.	Ban Methi	Shrub	Leaves	Nervous system disorder	CNS Depressant	[13, 214]
	*Sida cordata* (Burm.f.) Borss.Waalk.	Junka	Forb/herb	Leaves	Nervous system disorder, heat stroke	NRE	[215]
	*Sida cordifolia* L.	Berela	Subshrub	Leaves, bark of roots	Nervous debility	Parkinson’s disease	[173, 216]
Marantaceae	*Maranta arundinacea* L.	Ararut	Forb/herb	Rhizome	Epilepsy	NRE	[93]
Marsileaceae	*Marsilea minuta* L.	Shusni Shak	Forb/herb	Leaves, whole plant	Epilepsy, insomnia	Improve memory and learning	[106, 217, 218]
Melastomataceae	*Oxyspora cernua* Hook. f. & Thomson	Chokha	Herb	Leaves, root	Mental disorder	NRE	[101]
Menispermaceae	*Stephania japonica* (Thunb.) Miers	Akanadi	Climber	Leaves	Paralysis (Ch), vertigo, mental disorder	Antinociceptive	[13, 97, 160, 219, 220]
	*Tinospora crispa* (L.) Hook.f. & Thomson	Gulancha	Climber	Leaves, stem	Paralysis	Cerebral malaria	[118, 221]
Moraceae	*Ficus auriculata* Lour.	Kani-bot	Tree	Root	Epilepsy	NRE	[101]
	*Ficus hispida* L.f.	Dumur	Tree	Leaves, flower, seed, root, bark	Epilepsy, paralysis	CNS stimulation	[101, 222]
	*Ficus benghalensis* L.	Bot	Tree	Aerial root, bark	Epilepsy	Antinociceptive	[93, 223]
	*Ficus hederacea* Roxb.	Dumur	Shrub	Fruit, leaves	Epilepsy, paralysis	NRE	[99]
	*Ficus hirta* Vahl	Pakur	Shrub	Leaves, root	Schizophrenia	NRE	[4]
	*Ficus religiosa* L.	Pipal	Tree	Leaves, bark	Insanity	Memory deficit, Anti-Parkinson,	[113, 224, 225]
Moringaceae	*Moringa oleifera* Lam.	Sajina	Shrub	Leaves, fruit	Epilepsy (Sa), paralysis	CNS depressant, neuroprotective, dementia	[97, 106, 131, 226, 227]
Musaceae	*Musa sapientum* L.	Acchi-mio-bong (Ra)	Forb/herb	Leaves, stem	Memory loss	Acetylcholinesterase inhibition	[106, 228]
Nelumbonaceae	*Nelumbo nucifera* Gaertn.	Rakta padma	Forb/herb	Whole plant	Nervous debility	Memory impairment and brain damage	[106, 229]
Nyctaginaceae	*Boerhavia repens* L.	Punarnava	Herb	Leaves, whole plant, root	Epilepsy	NRE	[91, 97]
Oleaceae	*Jasminum sambac* (L.) Aiton	Bely Phul	Vine	Root	Insanity	Antidepressive and modulate mood in humans	[119, 230, 231]
Ophioglossaceae	*Helminthostachys zeylanica* (L.) Hook.	Shada Dhekia	Herb	Rhizome	headache (Kh)	Reduce inflammation of brain cells	[99, 232]
Orchidaceae	*Cymbidium aloifolium* (L.) Sw.	Tosabak, Suri mach (Ta)	Herb	Whole plant	Paralysis	Antinociceptive	[233, 234]
	*Rhynchostylis retusa* (L.) Blume	Tosabak	Herb	Whole plant	Epilepsy, vertigo	NRE	[234]
	*Vanda tessellata* Hook. *(*Syn:*Vanda roxburghii* R.Br.)	Rasna	Epiphytic herb	Aerial roots	Nervous system disorder	Anticholinesterase activity	[234–236]
Pandanaceae	*Pandanus foetidus* Roxb.	Keya kanta	Shrub	Root	Nervous debility	CNS depressant	[14, 237]
Parmeliaceae	*Usnea longissima* Ach.	Shailaj gach	Tree	Root, leaves	Nervous debility	NRE	[238]
Passifloraceae	*Adenia cardiophylla* Engl.	Pindopata	Tree	Bark, root	Headache, vertigo	NRE	[101]
	*Passiflora foetida* L.	Jhumkoludhi (Ch)	Climber	Leaves	Headache (Ch)	Epilepsy	[84, 252]
Phyllanthaceae	*Phyllanthus emblica* L.	Amloki	Tree	Fruit	Epilepsy (Tr), paralysis, headache	Alzheimer's disease, memory enhance	[88, 149, 239, 240]
	*Phyllanthus reticulatus* Poir.	Chitki, Panjuli, Chitkidari (Sa)	Shrub	Root, leaves	Epilepsy, heat stroke	Alzheimer's disease, Cognitive dysfunction	[113, 241, 242]
Piperaceae	*Piper betel* Blanco	Pan	Climber	Whole plant	Mental disorder (Be)	Cognitive dysfunction	[91, 165, 243]
	*Piper cubeba* L.f.	Kabab chini	Shrub	Fruit	Headache, mental disorder	Acetylcholinesterase inhibitor	[91, 107, 244]
	*Piper longum* L.	Pepul	Vine	Leaves, root	Paralysis	Neuroprotective, Parkinson's disease	[91, 245, 246]
	*Piper peepuloides* Roxb.	Pipil	Shrub	Leaves	Nervous debility	NRE	[98]
	*Piper retrofractum* Vahl	Choi	Climber	Leaves	Schizophrenia	Neurotrophic Activity, Alzheimer’s disease	[4, 247]
Plantaginaceae	*Bacopa monniera* (L.) Wettst.	Brammishak	Herb	Whole plant	Brain disorder (mental disorder), (Be), mental peace, insomnia, epilepsy	Memory enhance, Alzheimer's disease, neuroprotective	[91, 93, 219, 248, 249]
	*Scoparia dulcis* L.	Misridana	Subshrub	Root, fruit	Nerve system disorder	Increase memory	[14, 88, 177, 250]
Plumbaginaceae	*Plumbago auriculata* Lam.	Nil Chita	Shrub	Root, bark	Epilepsy, headache (Ga)	NRE	[127]
	*Plumbago rosea* L.	Lal Chita	Shrub	Root	Paralysis, memory loss	NRE	[88, 104]
Poaceae	*Cymbopogon citratus* Stapf	Dhan shabang	Herb	Leaves	Headache	Sedative, anxiolytic, hypnotic, neuroprotective	[251–253]
Polygonaceae	*Persicaria hydropiper* (L.) Delarbre	Bishkatal, Jiyoto (Sa)	Herb	Whole plant	Epilepsy	Acetylcholinesterase inhibitor	[241, 254]
	*Drynaria quercifolia* (L.) J.Sm.	Pankhiraj	Fern	Rhizome	Epilepsy (Ta), vertigo	Antinociceptive	[89, 212, 255]
Primulaceae	*Maesa indica* Wall.	Sesu, Sirkhi	Shrub	Whole plant	Paralysis	NRE	[99]
Ranunculaceae	*Nigella sativa* L.	Kalojira	Forb/herb	Fruit	Epilepsy	Alzheimer’s, Parkinson’s, schizophrenia	[93, 256, 257]
Rhamnaceae	*Gouania tiliifolia* Lam.	Moshkantur	Shrub	Leaves	Headache	NRE	[141]
	*Ziziphus mauritiana* Lam. (*Ziziphus jujube* Mill.)	Boroi	Tree	Leaves	Headache	Epilepsy, anxiolytic and hypnotic-sedative	[98, 99, 258–260]
Rubiaceae	*Borreria articularis* F.N.Williams	Todargil shak	Herb	Whole plant	Headache	NRE	[179]
	*Ceriscoides campanulata* Roxb.	Behlom	Tree	Leaves, fruit	Brain tonic (Nervous debility)	NRE	[261]
	*Hedyotis scandens* Roxb.	Bishlata	Climber	Whole plant	Paralysis, vertigo	NRE	[99]
	*Ixora cuneifolia* Roxb.	Beophul	Shrub	Leaves, root	Epilepsy	NRE	[99]
	*Ixora nigricans* R.Br. ex Wall.	Kalashona	Small tree	Leaves	Paralysis (Ch)	NRE	[129]
	*Maesa ramentacea* A.DC.	Moricha	Shrub	Leaves, root	Paralysis	NRE	[101]
	*Morinda angustifolia* Roxb.	Rang gach	Tree	Root, leaves	Epilepsy	NRE	[252]
	*Morinda citrifolia* L.	Holdi Kachu, Noni	Tree	Fruit, leaves	Schizophrenia	Stress-induced neurological disorder, prevent ischemic neuronal damage	[4, 66, 262]
	*Mussaenda roxburghii* Hook.f.	Ranirtak	Shrub	Root	Paralysis, epilepsy, headache (Ma)	NRE	[89, 252]
	*Ophiorrhiza mungos* L.	Gandhanakuli	Herb	Leaves, root	Mental disorder, paralysis	NRE	[89, 129]
	*Paederia foetida* L.	Gandal	Vine	Leaves	Paralysis (Sa)	NRE	[199]
	*Randia dumetorum* (Retz.) Poir.	Monkata	Shrub	Bark	Schizophrenia	NRE	[4]
Rutaceae	*Aegle marmelos* (L.) Correa	Bel	Tree	Leaves, fruit	Memory loss, schizophrenia, paralysis	Anticholinesterase activity	[4, 14, 88, 263]
	*Citrus grandis* Osbeck	Jambura	Tree	Fruit	Epilepsy	Memory enhance	[113, 264]
	*Clausena heptaphylla* Wight & Arn.	Alkatra (Ch), Pan mouri	Shrub	Fruit	Headache (Ch), mental disorder, epilepsy	NRE	[101, 129]
Santalaceae	*Santalum album* L.	Sheto chandan	Tree	Stem	Mental disorder, epilepsy, headache	Sedative	[93, 107, 108, 265]
Smilacaceae	*Smilax zeylanica* L.	Kumarialata	Climber	Leaves, stem	Memory loss	NRE	[146]
Solanaceae	*Datura metel* L.	Dhutura	Shrub	Leaves, flower, seed	Insanity, schizophrenia, mental disorder (Be)	Acute psychoactive	[4, 14, 91, 104, 266]
	*Solanum indicum* L.	Pokhongkhesi (Ma)	Herb	Fruit	Headache (Ma)	Protect blood–brain barrier breakdown	[252, 267]
	*Solanum torvum* Sw.	Tit Begun	Shrub	Fruit, leaves, root	Paralysis, insomnia	Anticonvulsant, antidepressant, anxiolytic	[113, 268, 269]
	*Withania somnifera* (L.) Dunal	Aswagandha	Undershrub	Whole plant	Mental disorder (Be)	Alzheimer’s disease, Parkinson’s disease	[79, 91, 270]
Stemonaceae	*Stemona tuberosa* Lour.	Lalguraniya alu	Herb	Tuber	Mental disorder	NRE	[179]
Taccaceae	*Tacca integrifolia* Ker. Gawl	Bara hikand	Herb	Tuber	Epilepsy, paralysis	NRE	[101]
Thymeliaceae	*Aquilaria agallocha* Roxb.	Agor	Tree	Wood	Nervous debility, headache	NRE	[114, 177]
Trapaceae	*Trapa natans* var. *bispinosa* (Roxb.) Makino	Panifol	Herb	Flower	Nervous debility	NRE	[101]
Urticaceae	*Boehmeria glomerulifera* Miq.	Borthurthuri	Shrub	Leaves	Epilepsy	NRE	[101]
	*Boehmeria kurzii* Hook.f.	Barokurzi	Shrub	Leaves, stem	Epilepsy	NRE	[101]
	*Elatostema papillosum* Wedd.	Silajhara	Herb	Leaves	Paralysis	Anticholinesterase activity	[101, 271]
	*Pouzolzia zeylanica* (L.) Benn.	Aguni-bolla gach	Herb	Leaves, root	Paralysis (Tr)	NRE	[149]
	*Sarcochlamys pulcherrima* Gaudich	Korobi	Shrub	Leaves	Paralysis	NRE	[101]
Verbenaceae	*Lantana camara* L.	Chotra,	Tree	Leaves	Headache (Ma)	Anxiolytic	[99, 272]
	*Phyla nodiflora* (L.) Greene	Saitta okra	Herb	Whole plant	Nervous system disorder	NRE	[273]
Vitaceae	*Cissus adnata* Roxb.	Bhatia-lota, Bodlar (Sa)	Climber	Stem	Paralysis (Sa), mental disorder, epilepsy, paralysis	Antinociceptive	[176, 274]
	*Cissus assamica* Craib	Amasha lata	Climber	Leaves	Mental disorder, paralysis	NRE	[101]
	*Cissus carnopa* Lam.	Gai goblae	Climber	Leaves, stem	Headache	NRE	[141]
	*Cissus javana* DC.	Rangila lata	Climber	Leaves, stem, root	Mental disorder	NRE	[101]
	*Cissus repens* Lam.	Marmaria Pata	Climber	Leaves	Epilepsy, vertigo	Antinociceptive	[89, 275]
	*Leea indica* Merr.	Bonfotka	Shrub	Leaves, root	Epilepsy	Sedative and anxiolytic	[101, 276]
	*Leea macrophylla* Roxb.	Hastikarma	Shrub	Leaves	Brain and nervous debility	NRE	[113]
	*Tetrastigma bracteolatum* (Wall.) Planch	Khurangul ludi		Leaves	Headache (Ch)	NRE	[129]
Xanthorrhoeaceae	*Aloe vera* L.	Ghritakumari	Herb	Leaves	Stroke, Paralysis	Protect neurotoxicity	[113, 258, 277]
Zingiberaceae	*Alpinia conchigera* Griff	Khetranga	Herb	Rhizome	Headache, vertigo (Ma)	Antinociceptive	[99, 278]
	*Alpinia nigra* (Gaertn.) B.L.Burtt	Jangli ada	Herb	Stem, rhizome	Vertigo (Ta)	CNS depressant	[212, 279]
	*Amomum aromaticum* Roxb.	Elach	Herb	Fruit	Mental and nervous system disorders, epilepsy	NRE	[107]
	*Curcuma aromatica* Salisb.	Jangli Halud	Herb	Leaves, rhizome	Vertigo (Ta)	Anti- depressant	[212, 280]
	*Curcuma longa* L.	Halud	Forb/herb	Rhizome	Memory loss	Reduce memory loss, Parkinson’s disease	[14, 88, 281, 282]
	*Kaempferia galanga* L.	Chandumula	Herb	Rhizome	Headache, paralysis (Ch)	CNS depressant	[160, 283]
	*Zingiber zerumbet* (L.) Sm.	Bhul-changa	Herb	Rhizome	Paralysis (Ch)	NRE	[184]

NRE: no recorded experiment on CNS disorder; Tribal community in parentheses

*Ba* Bauri; *Be* Beideye; *Ch* Chakma; *Ga* Garo; *Kh* Khumi; *Ma* Marma; *Or* Oraon; *Ra* Rakhain; *Sa* Santal; *Ta* Tanchongya; *Tr* Tripura

The CNS is a complex and sophisticated system, and today, CNS disorders are categorized and treated considering critical single or multiple targets. The traditional healers, particularly herbal medicine practitioners, focus on a typical category of disease commensurate with their knowledge and experience rather than employing a specific single biomarker targeted therapy. However, this review highlights ethnobotanical together with the respective experimental records focused on broadly categorized CNS disorders. The reviewed plant species, as a group, have been recommended against almost all classical types of CNS disorders.

## Materials and Methods

### Search Strategy

A comprehensive literature study published in journals, books, and reports was performed to get a systematic overview about the medicinal plants used against CNS disorders in Bangladesh. Various electronic databases were searched, including Web of Science, SciFinder, PubMed, Science Direct, Scopus, Springer, Taylor & Francis online, Wiley online library, and Google Scholar. The following keywords were employed in combination with Bangladesh: brain, memory, CNS, neurological disorder, neurodegenerative disease, psychological disorder, medicinal plants, traditional plants, survey of medicinal plants, ethnobotanical survey, ethnomedicinal survey, and survey of plants acting on CNS.

### Study Selection and Data Extraction

All publications dealing with plant species effective against CNS disorder have been identified from all of the possible sources published until the end of July 2020. The search was limited to literature published in English. The name of the plant species responsible in the treatment of CNS disorders has only been extracted among all other uses and species. For the pharmacological evidence, articles presenting first-hand research information including clinical, pre-clinical, ex-vivo, and in-vitro studies were also part of the inclusion criteria.

## History and Present Status of Traditional Bangladeshi Medicine (TBM)

Bangladesh, a tropical South Asian country, harbors a huge range of biodiversity including numerous medicinal plant species due to its diverse landscape and pronounced seasonal diversity [11]. Large parts of Bangladesh are covered by tropical forests featuring heterogeneous ecologic conditions such as fertile alluvial lands, warm and humid climates. Bangladesh is home to a rich plant diversity with more than 5300 species of higher plants [12]. Around 80% of the population of Bangladesh use herbal medicines for their primary healthcare where plants used in traditional ethnomedicine constitute a major component [13]. Bangladesh is also home to 35 indigenous communities living in various, mostly hilly, remote areas of Bangladesh; these communities contribute about 2% to the total population of the country. Each of these communities has a diverse cultural background and practices their own traditional ethnomedicine for primary healthcare [14].

## Distribution of Plant Species and Their Taxonomy

**Fig. 1:**
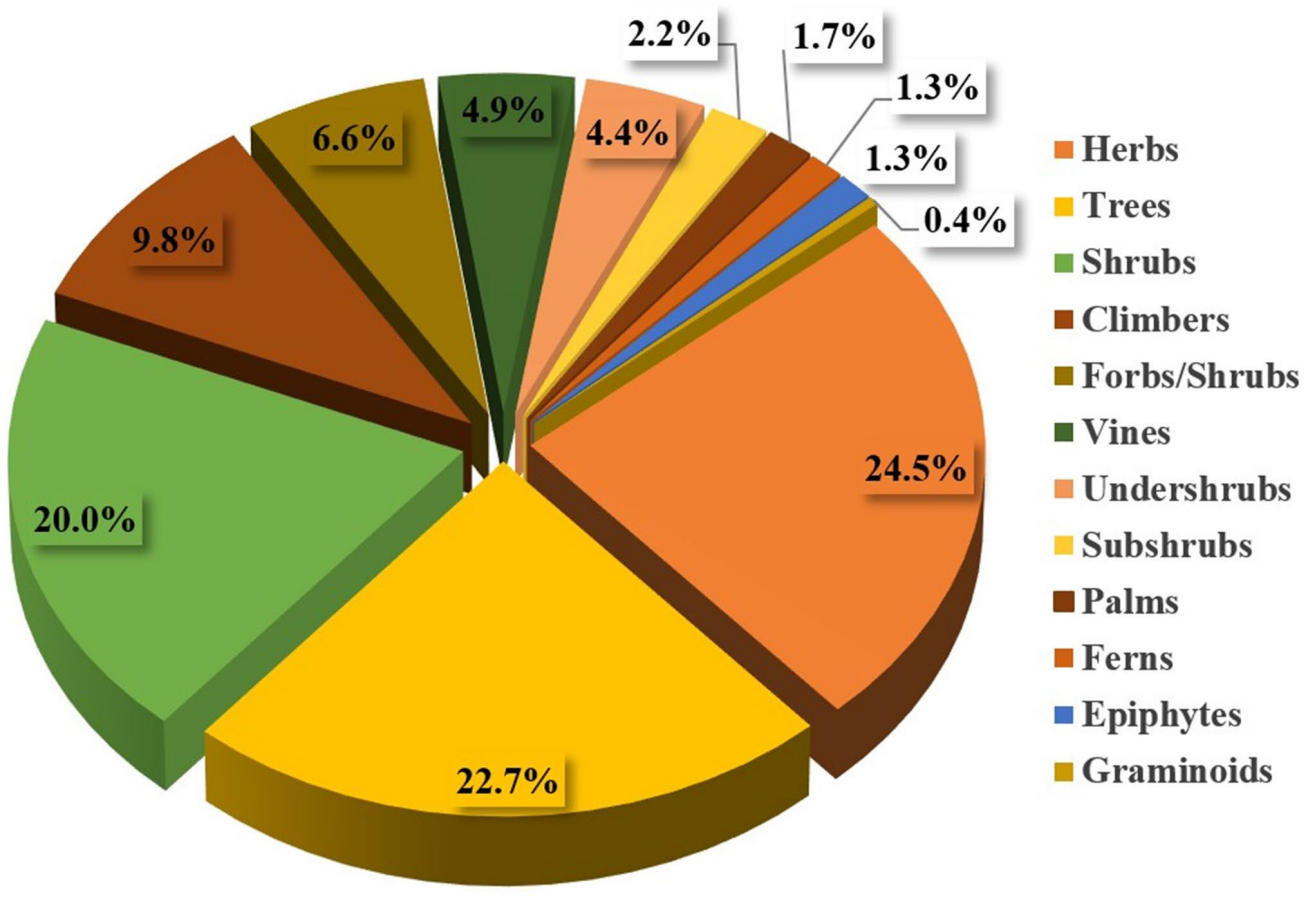
Growth habits of the covered species

A total of 224 plant species from 182 genera and from 81 different families were reported to be used against CNS disorders. All recorded plant species are presented in Table 1, detailing their family, local name(s), life-form, plant part(s) used, traditional uses, and the available pharmacological data supporting their traditional use. The life forms of the documented species were (in decreasing order) herbs (24.5%), trees (22.7%), shrubs (20.0%), climbers (9.8%), forbs/herbs (6.6%), vines (4.9%), undershrubs (4.4%), subshrubs (2.2%), palms (1.7%), ferns (1.3%), and epiphytes (1.3%) (Fig. 1). Analogous studies from other areas in tropical Asia yielded similar results regarding the life form of the medicinally used species [15–17].

**Fig. 2:**
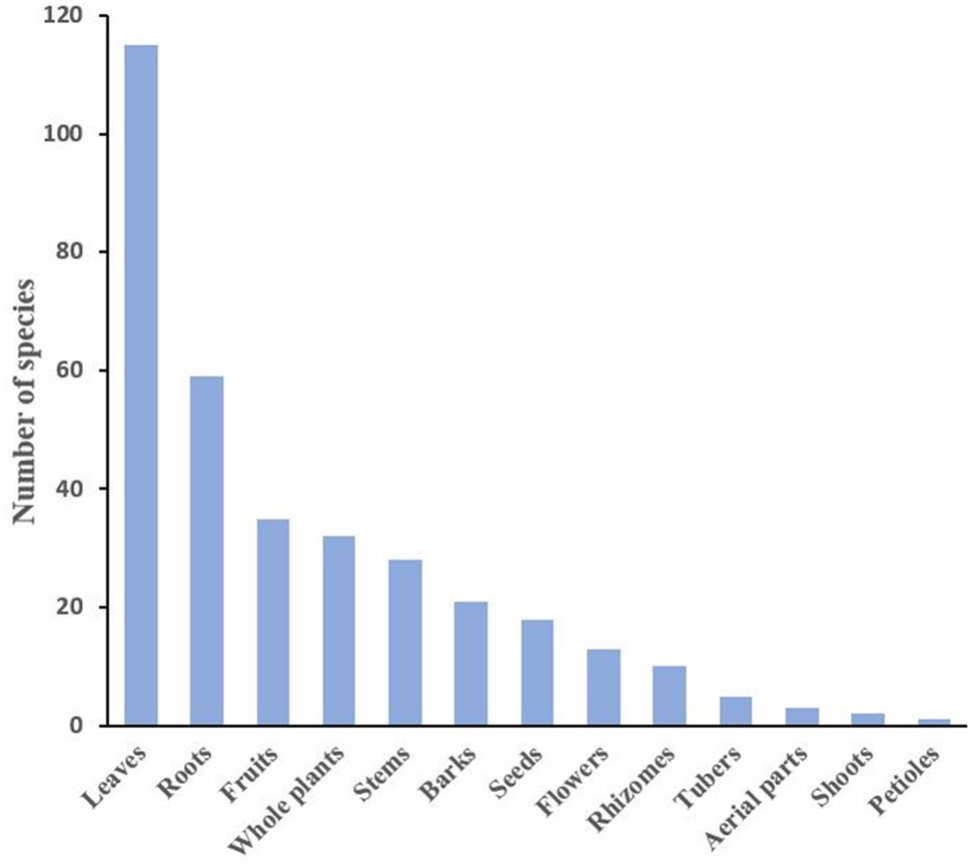
List of the most frequently used plant parts along with the number of corresponding species used in ethnomedicinal preparations

The most often utilized plant parts were leaves (51.3%), followed by roots (26.3%), fruits (15.6%), whole plants (14.2%), stems (12.5%), barks (9.3%), seeds, flowers, and rhizomes; while other parts were only rarely utilized (Fig. 2). Leaves are very often used in herbal medicine, because they often contain high amounts of active compounds and are easy to collect and prepare, and consequently, a larger number of plant natural product studies are available for leaves compared to studies concerning other plant organs. In our survey, roots were the second most frequently used plant organs, possibly due to their high concentration of bioactive compounds [18]. Regarding botanical systematics, the families with the highest number of species used against CNS disorders were the Fabaceae (syn.: Leguminosae; seventeen), Rubiaceae (twelve), Lamiaceae (ten), Apocynaceae, Cucurbitaceae and Vitaceae (each eight species), Euphorbiaceae, Malvaceae, and Zingiberaceae (each seven species), Araceae, Compositae, Fabaceae, Piperaceae, and Urticaceae (each five species), Amaranthaceae, Asteraceae, Moraceae, and Solanaceae (each four species). The remainder of the medicinally used plant families contributed only one to three species (Table 1).

According to the fundamental book on the Bangladeshi Flora [12], the largest five families in Bangladesh are the Poaceae, Fabaceae, Orchidaceae, Rubiaceae, and Asteraceae, respectively. The dominance of Fabaceae and Rubiaceae species in treating CNS disorder might amongst other factors, be explained by the presence of bioactive alkaloids, flavonoids, and terpenoids in many members of these families [19].

## CNS-Active Natural Products

Numerous plant natural products have been reported to have beneficial effects on the human CNS. Table 2 presents some of these natural products and their mechanism of actions. Two general postulates try to explain why natural products elicit effects on the human CNS: firstly, due to the connection of the numerous molecular signaling pathways that are conserved between the taxa and the systematic actions in natural product synthesis within plants [20]. The second hypothesis is that plant natural products exhibit similar effects on the nervous systems of humans and the most prevalent natural herbivores, via the same mechanisms [21].

**Table 2:** Bioactive compounds against CNS disorders from native species of Bangladesh

Species name	Active compounds	Mechanism of action	Ailments	References
*Acorus calamus* L.	α-Asarone, β-asarone	Acetylcholiesterase inhibitor	Alzheimer’s disease, memory loss	[284]
*Bacopa monniera* (L.) Wettst.	Bacoside A3, bacopaside II, bacopasaponin C, bacopaside X	Inhibits β-amyloid (Aβ) and fibrilation	Alzheimer’s disease, memory loss	[41]
*Blumea balsamifera* DC.	Blumpenes A, B, C, and D	Inhibition of NO	Alzheimer’s disease	[158]
*Cannabis sativa* L.	Δ^9^-Tetrahydrocannabinol	Inhibits β-amyloid	Alzheimer’s disease	[285]
		Attenuates the motor coordination deficits and huntingtin aggregate	Huntington’s disease	[85]
		Prevent neuronal damage	Parkinson’s disease	[286]
	Cannabidiol	Reduces Aβ–induced neuroinflammation	Alzheimer’s disease	[287]
		Prevent neuronal damage	Parkinson’s disease	[286]
*Centella asiatica* (L.) Urb.	Asiatic acid	Prevent MPTP/p-induced neuronal cells loss	Parkinson’s disease	[47]
*Citrus grandis* Osbeck	3,5,6,7,8,3′,4′-Heptamethoxyflavone	Induce activation of ERK1/2 and CREB in cultured neurons	Memory disorders, Alzheimer’s disease	[264]
*Clerodendrum infortunatum* L. (Syn:*Clerodendrum viscosum* Vent.)	Acteoside	Inhibits β-amyloid	Alzheimer’s disease, cognitive deficit	[82, 83]
*Costus speciosus* (J.Koenig) Sm.	Costunolide	Inhibition of NFkappaB and MAPKinase activation	Neuroinflammatory diseases	[167]
*Curcuma longa* L.	Curcumin, demethoxycurcumin, bis-demethoxycurcumin	Prevent acute neuroinflammation, mitochondrial dysfunction and apoptosis	Neuroinflammation and memory impairment, Parkinson’s disease	[50, 51]
*Cyperus rotundus* Vahl	α-Cyperone, terpinen-4-ol	Destabilization of microtubule fibers in brain	Brain inflammation	[61, 62]
*Lantana camara* L.	Ursolic acid stearoyl glucoside	Unknown	Anxiety	[272]
*Nigella sativa* L.	Thymoquinone	Inhibits β-amyloid	Alzheimer’s disease	[288]
		Retrieved dopaminergic neurons	Parkinson’s disease	[257]
*Oroxylum indicum* (L.) Benth.	Apigenin, baicalein, baicalin, chrysin, hispidulin, oroxylin A	Induce neuronal differentiation	Disorder of nerve tissue development	[144]
*Piper betel* Blanco	Hydroxychavicol	Atenuate cytokines and both β- and γ-secretase	Cognitive dysfunction, Alzheimer’s disease	[243]
*Piper cubeba* L.f.	Cubebin	Acetylcholiesterase inhibitor	Cognitive dysfunction, Alzheimer’s disease	[244]
*Piper retrofractum* Vahl	Piperodione	Nerve growth factor (NGF) potentiation	Neurodegenerative diseases, Alzheimer’s disease	[247]
*Santalum album* L.	α-Santalol, β-santalol	Sedation	Anxiety	[289]
*Solanum indicum* L.	Seasmol	Protect blood–brain barrier breakdown	Alzheimer’s disease and multiple sclerosis	[267]
*Withania somnifera* (L.) Dunal	Withanolide A, withanone, withaferin A, withanoside IV, sitoindoside VII, sitoindoside VIII, sitoindoside IX, sitoindoside X	Prevent loss of axons, dendrites, and synapses; neuroprotection, enhance antioxidant enzymes	Alzheimer’s disease	[73, 78, 80, 290]
*Ziziphus mauritiana* Lam. (Syn: *Ziziphus jujube* Mill.)	*cis*-9,10Octadecenamide, jujuboside-A, jujuboside-B	Increase cholinesterase and cholinesterasetrasferase activity, GABA-binding modulation	Epilepsy, depression, memory loss	[259, 260, 291]

*CREB* cAMP response element-binding protein; *MAPKinase* mitogen-activated protein kinases; *ERK1/2* extracellular signal-regulated kinase-1/2; *NFkappaB* nuclear factor kappa of activated B cells

Alkaloids are one of the largest groups of plant natural products. These compounds usually act as agonists and antagonists to a variety of neurotransmitter through direct binding to neuro-receptors and/or by interference with neurotransmitter metabolism. Plant-derived alkaloids possess potential therapeutic effects against several neurodegenerative disorders (Alzheimer's disease, Huntington's disease, and Parkinson's disease), epilepsy, schizophrenia, and stroke [22].

Phenols are the most widespread and ubiquitous class of natural products. Besides free radical and reactive oxygen species scavenging, and metal chelating abilities, phenolic compounds demonstrate a significant role in various CNS disorders by direct interaction with neurotransmitter systems including sedative, anxiolytic, antipsychotic, cognitive enhancement, cholinergic upregulation, and antidepressant effects [23].

Saponins are a structurally diverse group of glycosidic compounds, featuring either pentacyclic triterpenoids or steroids as aglycones. Saponins have significant neuroprotective effects on the attenuation of CNS disorders, such as stroke, Alzheimer's disease, Parkinson's disease, and Huntington's disease [24]. In this review, Table 2 displays a selection of saponins (Fig. 9), which are potentially effective on brain disorders. Terpenes are a large class of natural products exhibiting a wide range of effects within the CNS. Many natural terpenoids have been reported to interact with the octopaminergic and noradrenergic systems, to inhibit cholinesterase, and to directly or allosterically bind to the GABAergic system; all with a relation to disorders like anxiety, insomnia, convulsion, pain, and cognitive deficits [25].

## Plants, Traditional Medicines, and CNS Disorder: Globally

Approximately one out of nine human deaths is related to a nervous system disorder worldwide, and more than 28% have to live with disability caused by nervous system disorder at some stage of their lives [26]. Depression is the major cause of disability and is globally more frequent than all other nervous system disorders. The top twenty leading causes for disability also include anxiety disorders, schizophrenia, autism and Asperger syndrome, Alzheimer’s disease and other dementias, and illicit drug use [27].

In traditional systems of medicine, plants have been used to treat a huge number of disorders including nervous disorder for centuries, because they are easily available and affordable. The latest global survey of traditional and complementary medicine (T&CM) shows that significant momentum has been achieved over the past decade (WHO, 2013). Over 100 million Europeans are currently using T&CM. Thus, in Europe one fifth of the population regularly use T&CM and the same share is preferring healthcare, which includes T&CM [28]. It is evident that there are many more T&CM users in Africa, Asia, Australia, and North America [29]. Traditional medicines could be a potential source of novel compounds or phytomedicines/supplements in the management of nervous disorders. Apomorphine, galanthamine, lisdexamfetamine, and valproic acid (Fig. 4) are the first line drugs currently used to treat Parkinson’s disease, Alzheimer’s disease, attention-deficit/hyperactivity disorder, and epilepsy, respectively. The active compounds were originally derived from *Papaver somniferum* L, *Galanthus nivalis* L.*, Ephedra sinica* Stapf., and *Valeriana officinalis* L., respectively. Since the 1950s, the FDA approved six plant derived drugs (Fig. 4), namely benzatropine (1954) (derived from atropine from e.g. *Atropa belladonna* L.), levodopa (1970) [from *Mucuna pruriens* (L.) DC.], carbidopa (1975) (from levodopa, e.g. from *Mucuna pruriens*), pergolide (1988) [from ergot alkaloids from, e.g. *Claviceps purpurea* (Fr.) Tul.], melevodopa (1993) (from levodopa from, e.g. *Mucuna pruriens*), and apomorphine (2004) (from morphine from e.g. *Papaver somniferum*) to treat Parkinson’s disease. A report showed that by the end of 2013, the FDA had approved 307 natural products and natural product derivatives from plants, bacteria, fungi, and marine organisms, respectively. These comprise 21% of all approved new chemical entities [30].

## Plants, Traditional Medicines, and CNS Disorder: in Bangladesh

In a global study, Bangladesh has been ranked 133^rd^ among 195 countries regarding personal healthcare access and quality [31]. In Bangladesh, over six million people experience depressive disorders and almost seven million people are suffering from anxiety disorders [32]. It is estimated that more than ten thousand people are dying every year by suicide in the country [33]. Most of the nervous system disorders are chronic and polygenic in nature. The development of more effective treatments, for example in schizophrenia and depression, based on selective drugs for single molecular targets has been largely unsuccessful [34]. Hence, multi-targeted therapeutic approach of nervous system disorders employing traditional medicine is often advantageous, easier, cheaper, and more cost effective. A handful of ethnomedicinal surveys on medicinal plants over different divisions, districts, villages, and even hill tract and tribal areas of the country revealed that medicinal plants are used to treat various disorders including nervous system disorders. Among the medicinal plants used in nervous system disorders Sotamuli (*Asparagus racemosus*), Thankuni (*Centella asiatica*), Akanadi (*Stephania japonica*), Bel *(Aegle marmelos*), Telakucha (*Coccinia grandis*), Tagar (*Tabernaemontana divaricate*), Misridana (*Scoparia dulcis*), Brammishak (*Bacopa monnieri*), and Aswagandha (*Withania somnifera*) are the most popular herbal medications for nervous system disorders in Bangladesh (Table 1). Table 1 contains all local names of the plant species used against CNS disorders in Bangladesh.

**Fig. 3:**
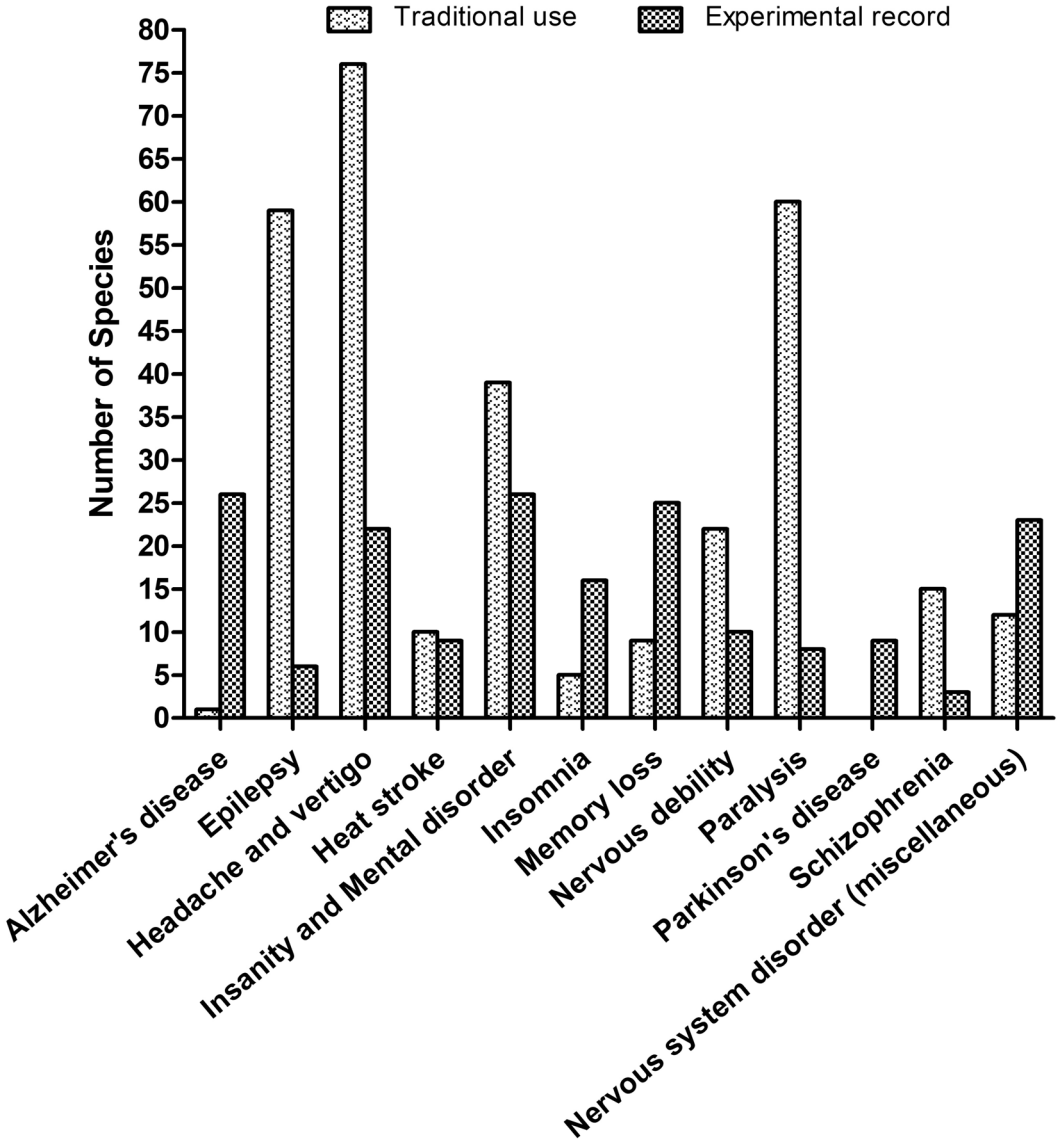
Comparison of the documented plant species with traditional use and experimental evidence over categorized CNS disorders

Ulothkombal (*Abroma augusta*), Apang (*Achyranthes aspera*), Halud (*Curcuma longa*), Noni (*Morinda citrifolia*), Sajina (*Moringa oleifera*), and Mutha (*Cyperus rotundus*) are also widely used in the management of CNS disorders. All of the above-mentioned species have demonstrated their notable pharmacological activity against nervous system disorders in different experimental models. The experimental evidence available for *Achyranthes aspera*, *Aegle marmelos*, *Asparagus racemosus*, *Bacopa monnieri*, *Bryophyllum pinnatum*, *Centella asiatica*, *Clitoria ternatea*, *Coccinia grandis*, *Convolvulus pluricaulis*, *Curcuma aromatica*, *Curcuma longa*, *Datura metel*, *Euphorbia neriifolia*, *Hemidesmus indicus*, and *Musa sapientum* also support the claims of traditional users (Table 1). In addition, for some of the species traditionally used in various nervous disorders, no pharmacological investigations have been performed yet, including Ghetkaachu (*Typhonium trilobatum*), Kundri (*Solena amplexicaulis*), Lal Chita (*Plumbago rosea*), Dheki shak (*Lygodium altum*), and Kanchan (*Bauhinia acuminata*) (Table 1). To cure paralysis, epilepsy, insanity and mental disorder, and nervous debility are the most often mentioned indications among all covered CNS disorders. In contrast, most experimental evidence so far has been provided for activity against insanity and mental disorder, memory loss, and Alzheimer’s disease (Fig. 3).

**Fig. 4:**
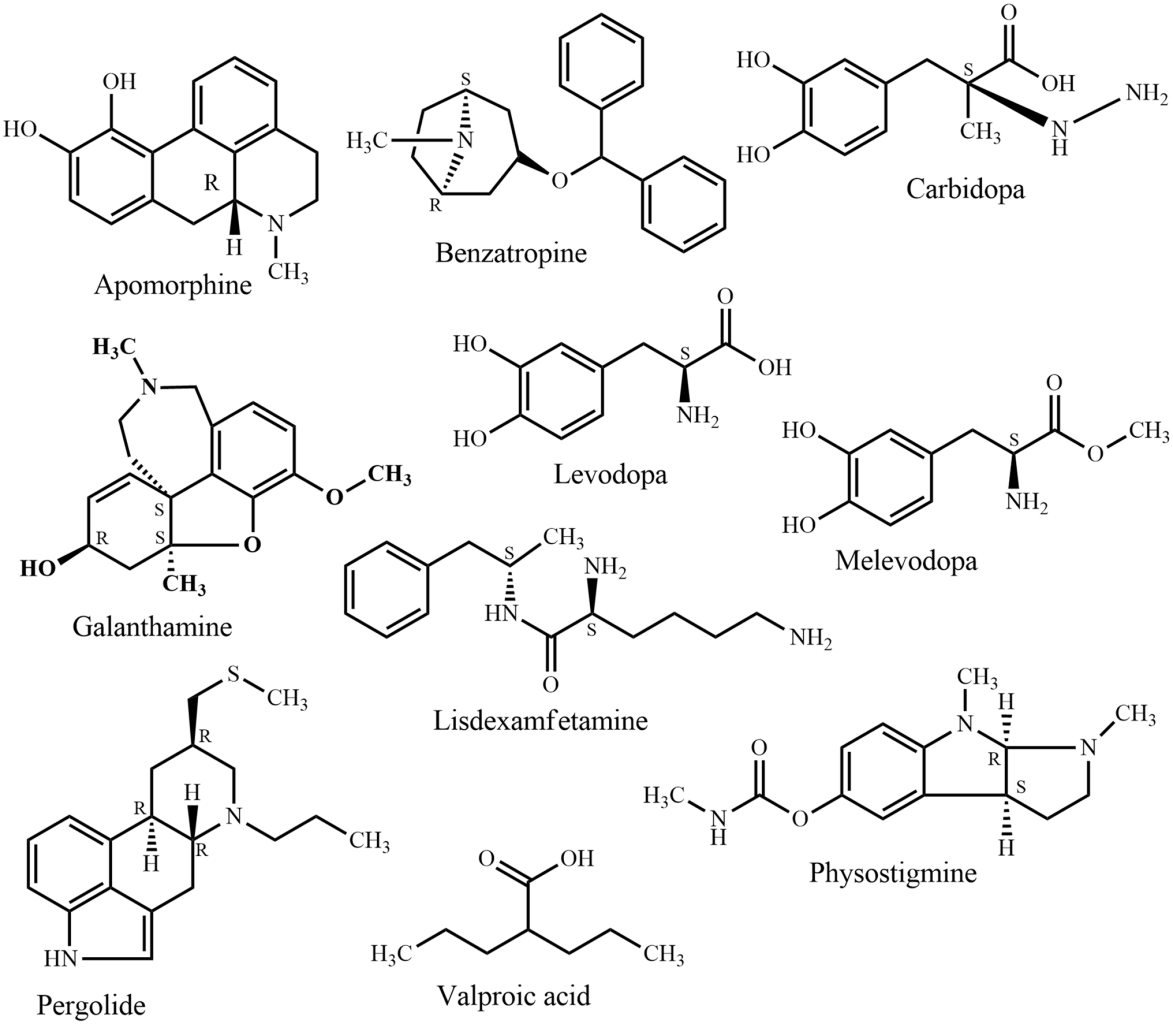
Chemical structure of some commonly used natural products for the treatment of nervous system disorders

Traditional medicine and conventional healthcare systems are offered in separate facilities at secondary and tertiary levels in nine countries in South-Eastern Asia (Bangladesh, Bhutan, India, Indonesia, Maldives, Myanmar, Nepal, Sri Lanka, and Thailand), while all three levels of care are available in the same health care facilities in South Korea. In Bangladesh, there are 469 small factories (268 Unani and 201 Ayurvedic) producing traditional drugs worth approximately US$ 100 million every year [35] (Fig. 4).

## Evidence-Linked Plants and Active Metabolites of TBM Effective on CNS Disorder

**Fig. 5:**
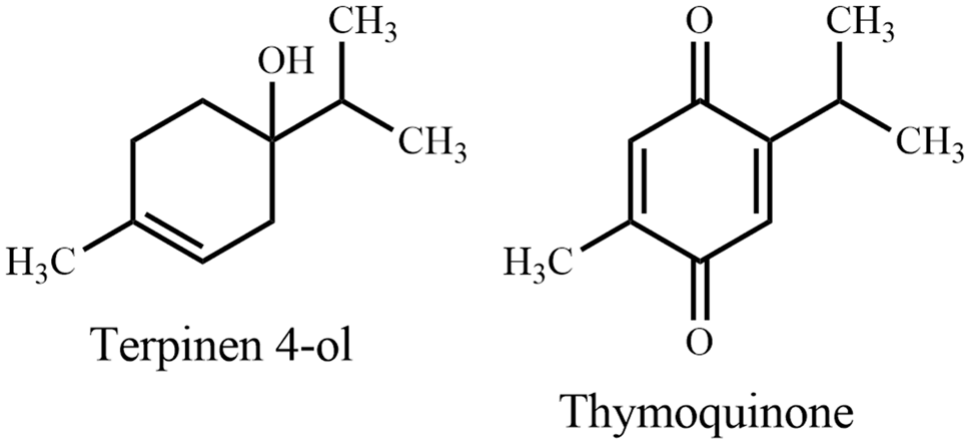
Monoterpenes

**Fig. 6:**
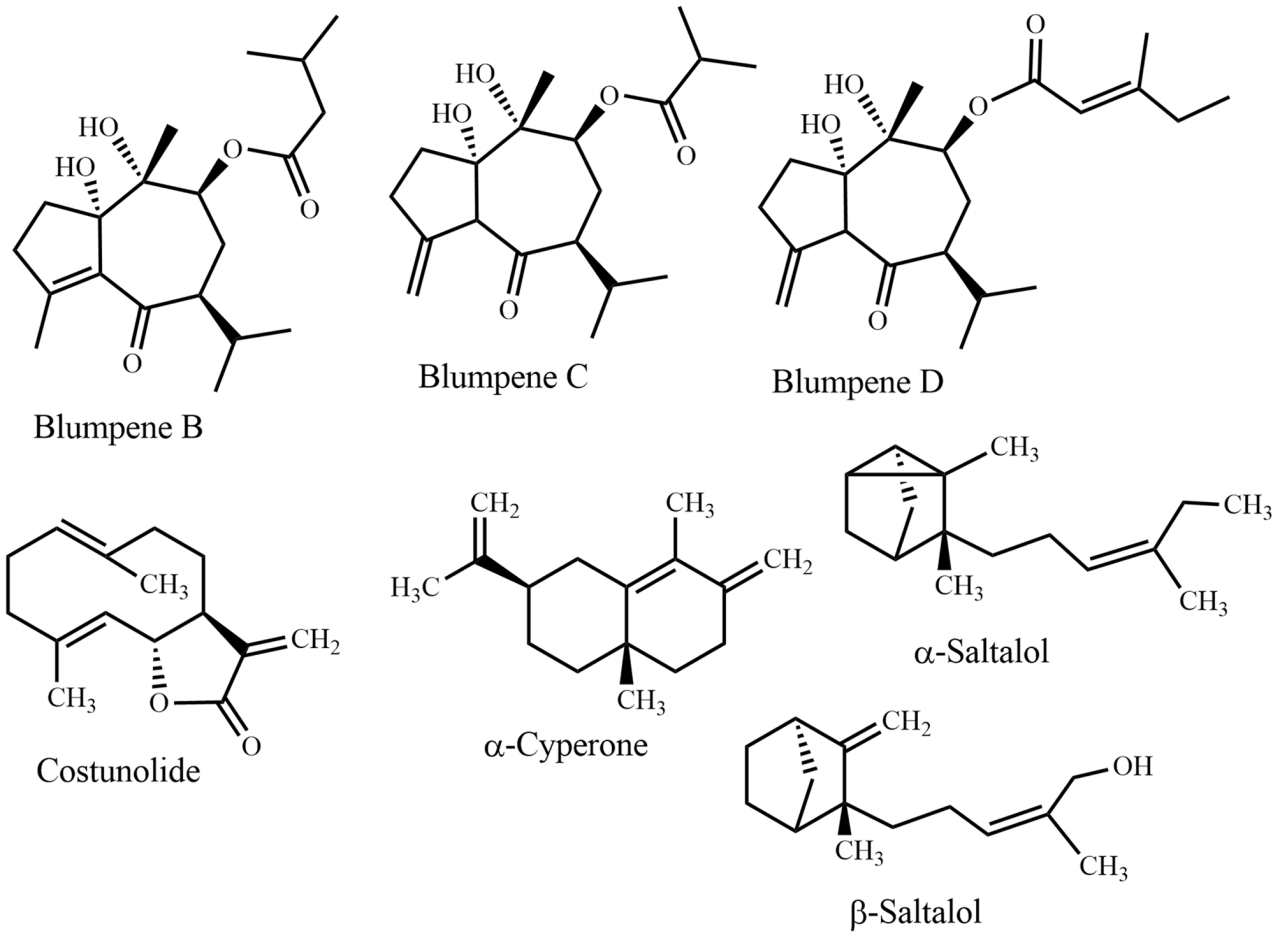
Sesquiterpenes

**Fig. 7:**
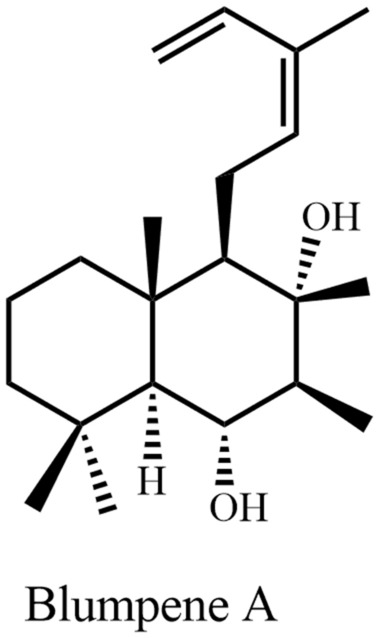
Diterpene

**Fig. 8:**
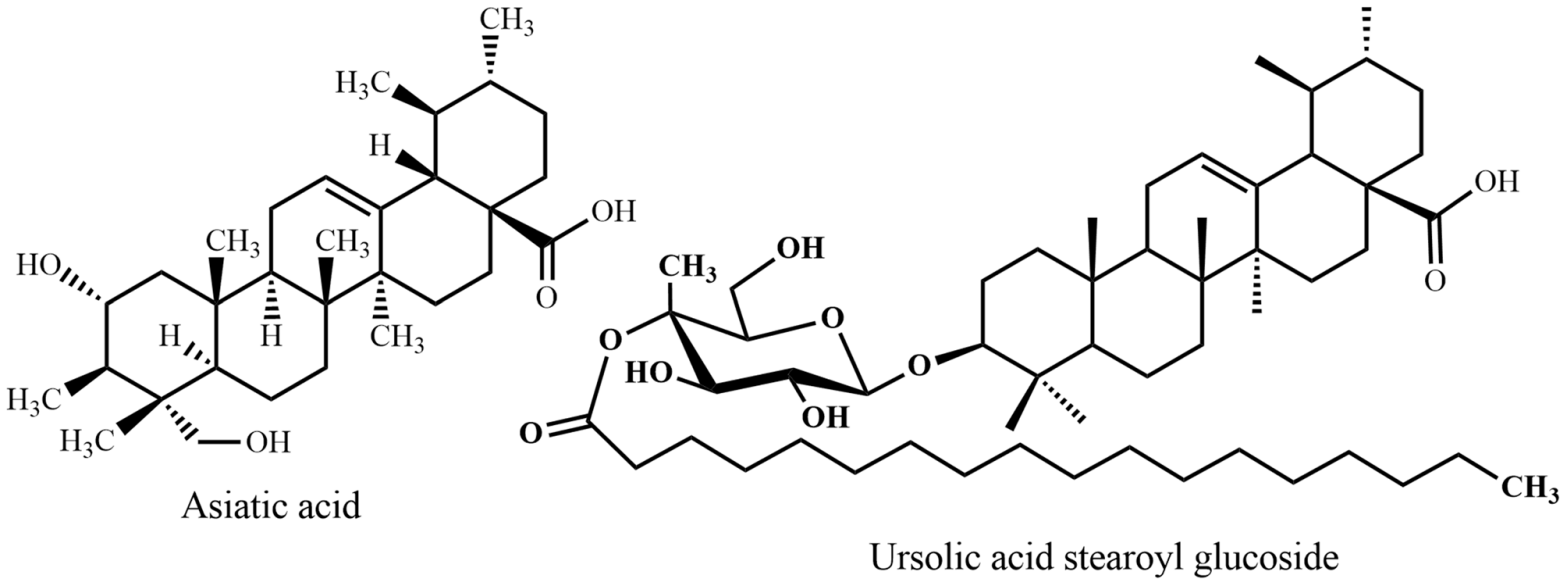
Triterpenes 1: ursane derivatives

**Fig. 9:**
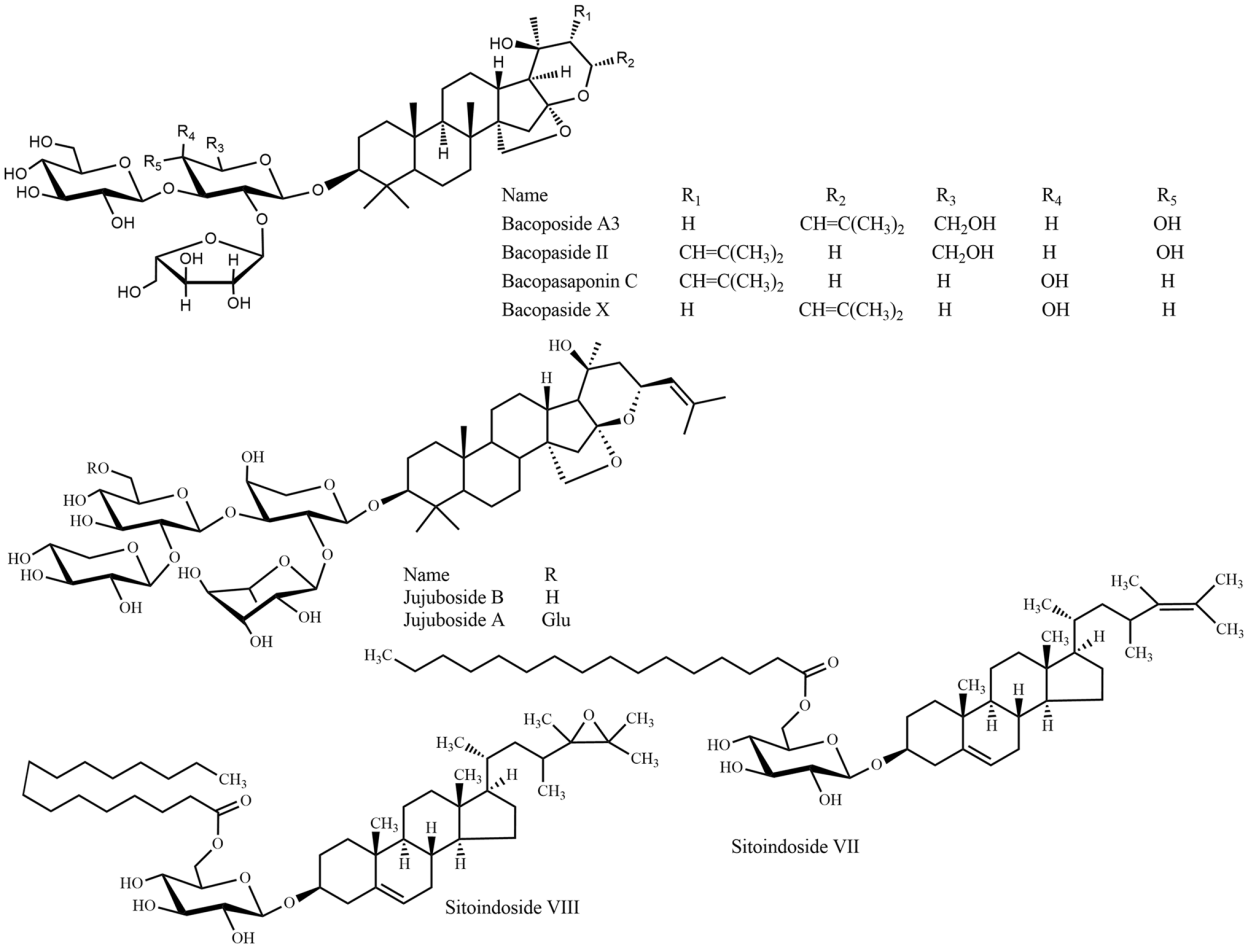
Triterpenes 2: steroidal saponins

**Fig. 10:**
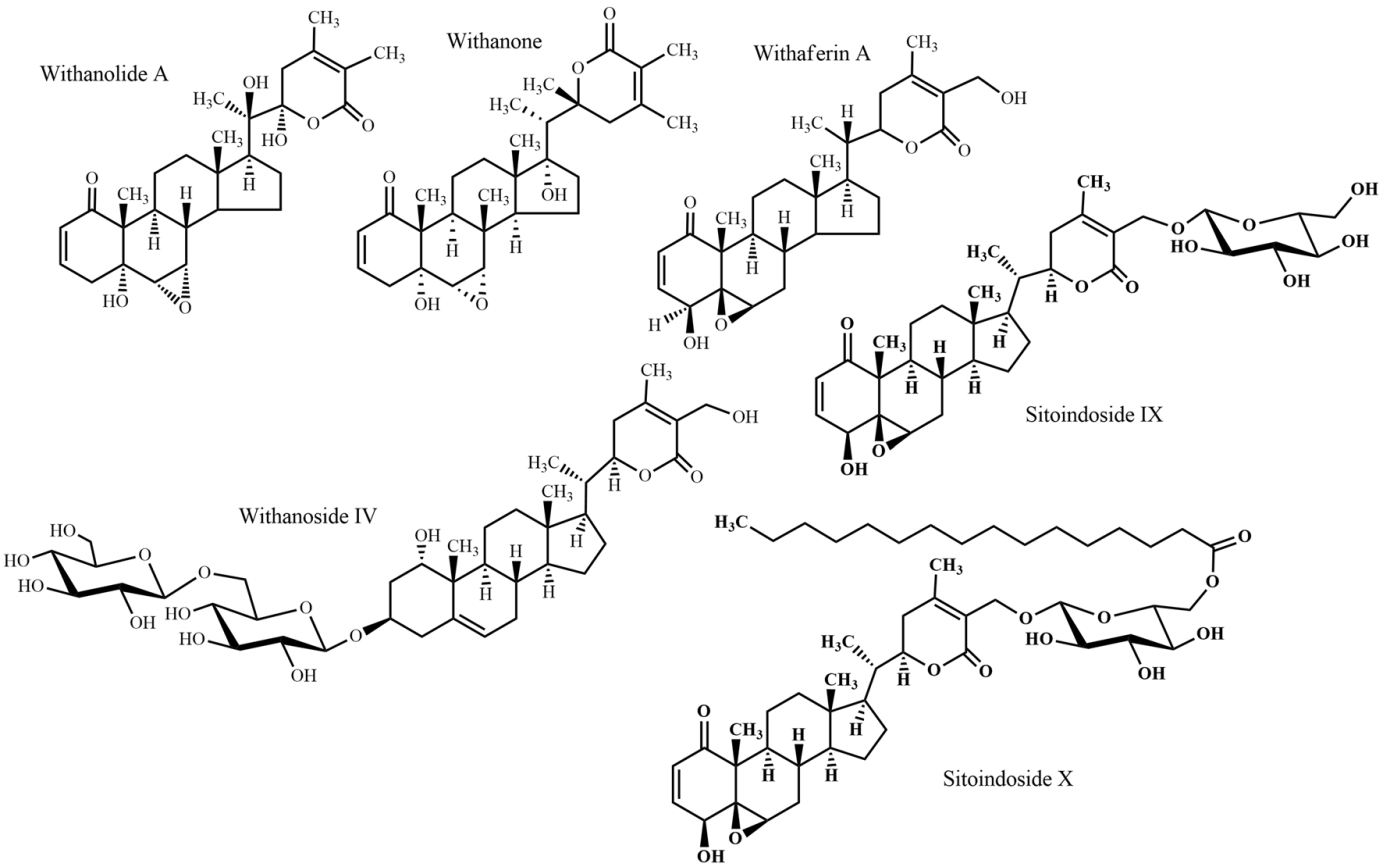
Triterpenes 3: steroidal lactones

**Fig. 11:**
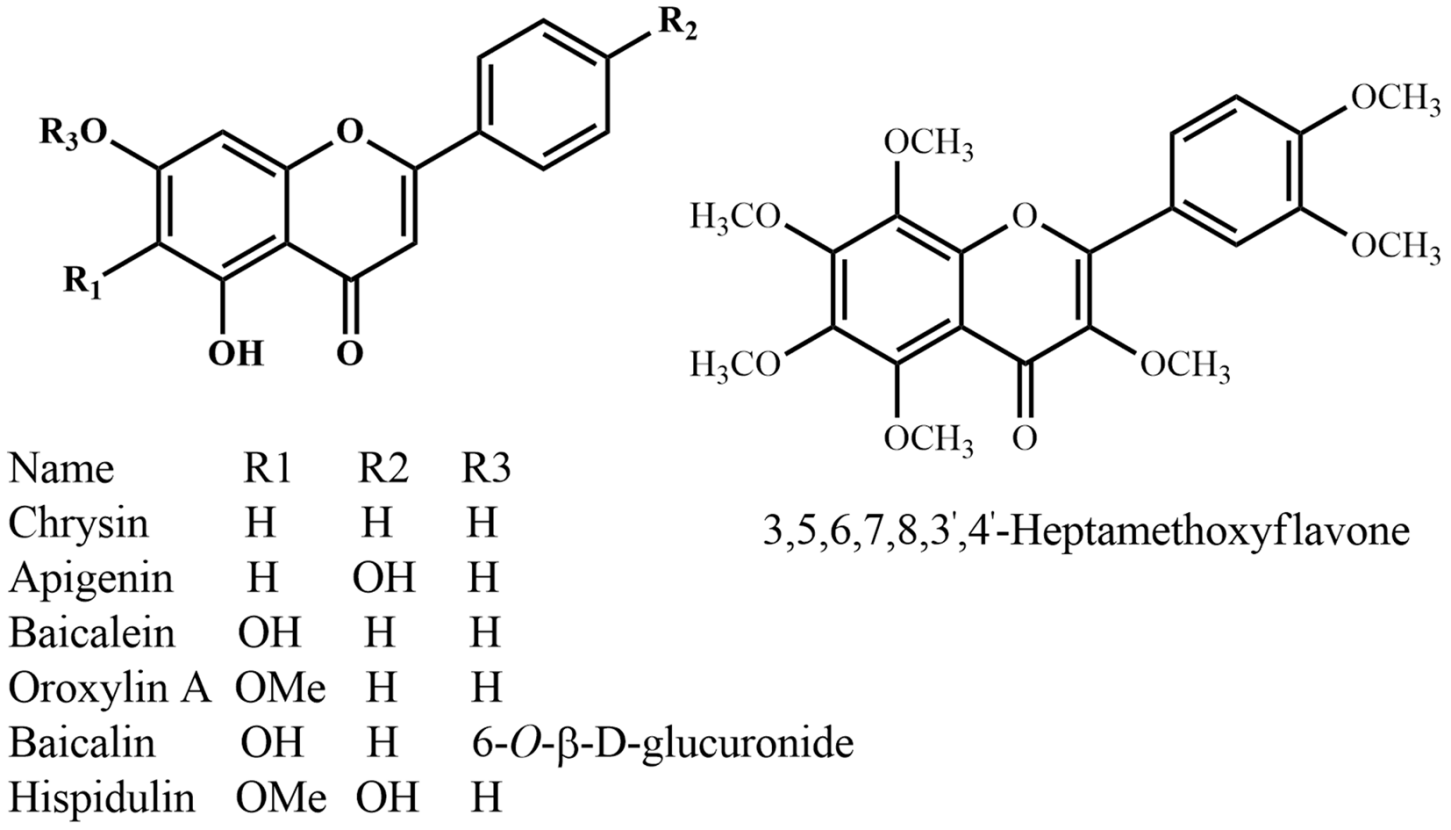
Flavonoids

**Fig. 12:**
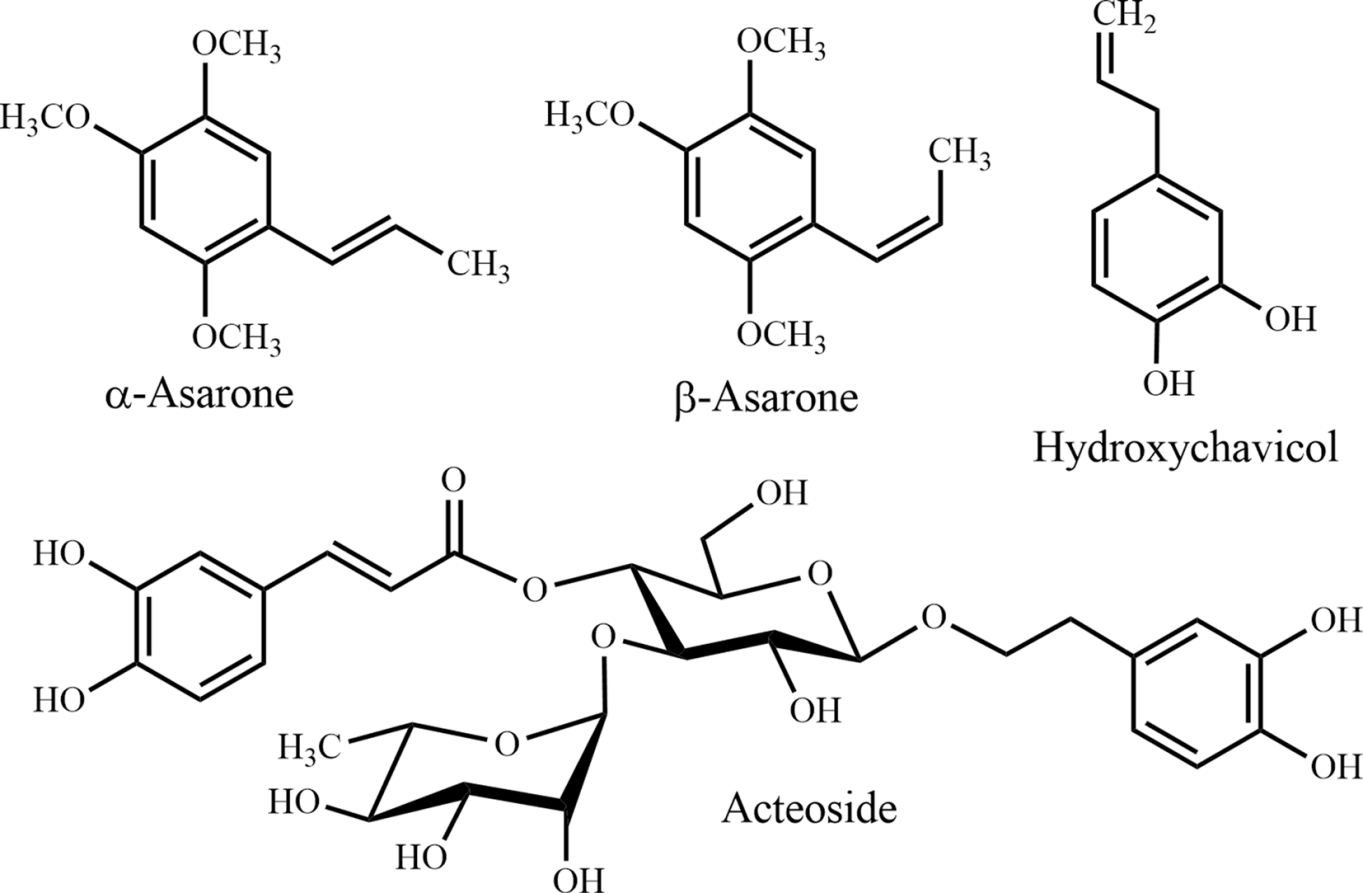
Phenylpropanoids

**Fig. 13:**
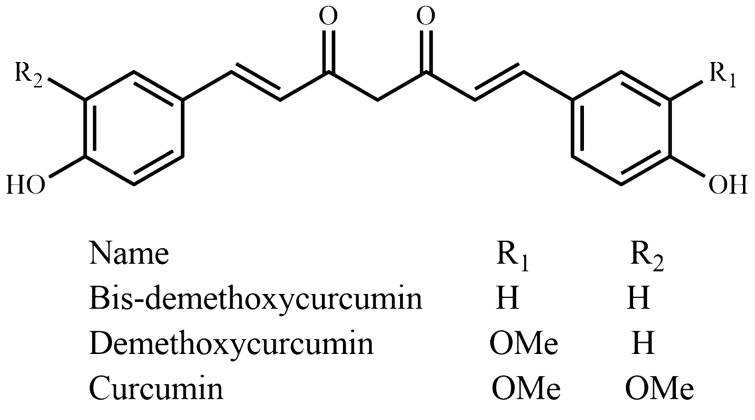
Diphenylheptanoids

**Fig. 14:**
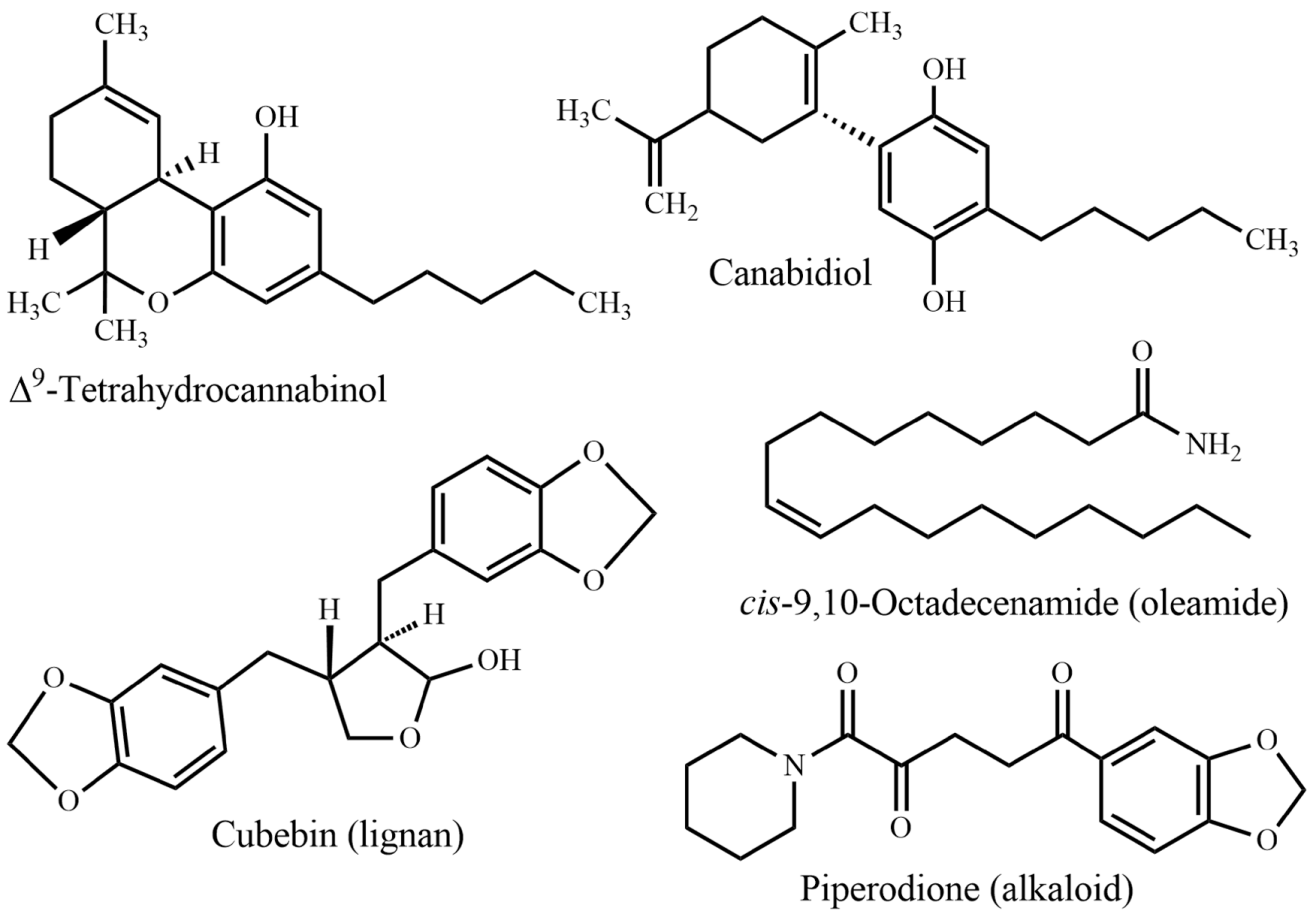
Miscellaneous (cannabinoid, lignan, oleamide, and alkaloid)

Many plant-derived natural products are claimed to have beneficial effects against CNS disorders. Some pure natural products derived from the plant species mentioned in this review, have already been tested as efficacious candidates against CNS disorders. Table 2 displays these metabolites with the corresponding disorder, where they were found to be active. Name and structures of all mentioned plant natural products from different source species have been summarized in Table 2 and in Figs. 5, 6, 7, 8, 9, 10, 11, 12, 13, and 14 (based on chemical compound classes). From all of the mentioned species, *Bacopa monnieri*, *Centella asiatica*, *Curcuma longa*, *Cyperus rotundus*, *Morinda citrifolia*, and *Withania somnifera* have been selected and discussed in some detail below. The focus of the discussion is on their impact on nervous system disorders. The species have been selected based on their widespread use, a large body of experimental records, and commercial availability. The main point of giving in-depth records on some selected species is to show the large potential of such traditional medicinal plants both from a medicinal and from a commercial perspective.

*Bacopa monnieri*, "Brammishak", a small herb from the Plantaginaceae family, is distributed mainly in the coastal area of Bangladesh such as Chittagong, Cox's Bazar, and Saint Martin's island. Brammishak is named after the word ‘Brama’, the mythical ‘creator’ in the Hindu pantheon. ‘Brahmi’, which also means ‘bringing knowledge of the Supreme Reality’ [36]. The herb was used by ancient Vedic scholars to sharpen the cognitive functions and is mentioned as part of many Ayurvedic preparations. Brammishak is also traditionally used as a green leafy vegetable (shak) due to its well-known health benefits [37]. The experimental evidence has proven potent activity of Brammishak on the regulation of reactive oxygen species, neuroprotection, acetylcholinesterase (AChE) inhibition, choline acetyltransferase activation, β-amyloid reduction, increased cerebral blood flow, and monoamine potentiation and modulation [38]. Brammishak contains triterpenoid saponins called bacosides. Among the twelve analogs of bacosides, bacoside A is the best studied and most potent constituent of Brammishak, which additionally includes bacoside A3, bacopaside II, bacopasaponin C, and bacopaside X (a jujubogenin isomer of bacosaponin C) (Fig. 9) [39]. Bacoside A significantly inhibit β-amyloid toxicity, fibrillation, improve memory and cognitive functions, decreased GABA receptors associated with epilepsy as well as increased the activities of superoxide dismutase, catalase, glutathione peroxidase, and glutathione reductase [40, 41]. In a review study on human trials, Neale et al. [42] compared the nootropic effects of two neutraceuticals Brammishak and *Panax ginseng* with modafinil (a synthetic eugeroic drug); in this comparison, Brammishak displayed the most consistent and largest effect of the three tested preparations.

*Centella asiatica*, "Thankuni", a perennial herbaceous creeper with kidney shaped leaves belonging to the Apiaceae family, is distributed throughout Bangladesh in fallow lands. Thankuni leaf is an ancient Ayurvedic, Unani, and has been used as a folk medicine in Bangladesh and South Asian countries for many centuries. The species is used as a revitalizing herb that supposedly strengthens nervous function and memory. An aqueous extract of *C. asiatica* leaves contributes to improved learning and memory processes by modulating dopamine, 5-hydroxytryptamine (5-HT), and noradrenaline systems in rat brains in vivo [43]. This result suggested that the polar compounds, for example asiatic acid present in *C. asiatica* leaves, may enhance cognitive functions by influencing neurotransmitter systems in the CNS. Further research proved that asiatic acid (triterpenoid) (Fig. 8) from *C. asiatica* down-regulates β-secretase (BACE1) as well as up-regulates ADAM10 in primary rat cortical neurons [44], inhibits induced neurotoxicity of aged rats [45], attenuates glutamate-induced cognitive deficiencies of mice, and protects SH-SY5Y cells against glutamate-induced apoptosis [46], which are all related to potential routes in Alzheimer’s disease treatment. Asiatic acid from *C. asiatica* effectively offered neuro-protection in chronic Parkinson’s disease by activation of dopaminergic neurons [47]. Orhan et al. [48] showed that butyrylcholinesterase inhibitory activity of South Asian *C. asiatica* is stronger than from Chinese sources.

*Curcuma longa*, "Halud", is a perennial rhizomatous herb from the Zingiberaceae family and is cultivated all over Bangladesh and used as one of the main spice. Along with the protection of memory loss, it contributes to a wide range of potential medicinal applications because of the presence of curcuminoids (Fig. 13). Curcumin, an extensively studied plant natural product isolated from the rhizome of *Curcuma longa* has displayed neuro-pharmacological activity against neuro-inflammation, memory impairment, and different biomarkers of Alzheimer’s disease and Parkinson’s disease in vitro and in vivo [49–51]. More importantly, curcumin has already been clinically evaluated against a few central nervous system disordersseases. Initially, Rainey-Smith et al. [52] reported a low efficacy of curcumin against dementia symptoms. However, recently developed novel curcumin formulations (Longvida® and Theracurmin) ensure a higher bioavailability, combined with good acute and chronic activities for both products, even at low doses (80–180 mg/day) [53]. The study carried out by Burns et al. [54] showed a marked improvement in a patient trial of Déjérine-Sottas disease, where curcumin was administered for twelve months in two escalating doses (1500 and 2500 mg/day). In the curcumin-treated group, it was observed that curcumin decreased IL-1β, TNFα, salivary cortisol levels, and increased plasma BDNF [55]. Lopresti et al. [56] identified a significant increase in urinary molecular markers thromboxane B2, substance P, baseline plasma endothelin-1, and leptin that can all be related to the antidepressant mechanism of action of curcumin.

*Cyperus rotundus*, "Mutha", a perennial herb as well as an obnoxious weed, is widely distributed in tropical and subtropical regions, including Bangladesh. This species traditionally used in the management of paralysis in Bangladesh, and epilepsy in India [57]. Additionally, experimental evidence showed a potential role in improving memory and cognition. Rhizomes of *C. rotundus* possess anti-AChE activity [58], anticonvulsant properties [59], inhibits memory loss [60] and pyramidal cell loss. Nóbrega et al. [61] reported that terpinen-4-ol (Fig. 5) (contained in the essential oil of *C. rotundus*) is effective against convulsion in behavioral and electrophysiological studies. Azimi et al. [62] identified α-cyperone from *C. rotundus* as capable of interactions with tubulin and as a destabilizing agent of microtubule polymerization. This interaction results in reduction of inflammation, which could be beneficial for the treatment of inflammatory diseases such as Alzheimer’s disease.

*Morinda citrifolia*, "Noni", a small tropical tree of the Rubiaceae family, is native to South Asia and cultivated all over Bangladesh [63]. All parts of the plant are claimed to have various pharmacological properties, in particular, the fruit has a long history of dietary use in tropical regions [64]. In 2002, Noni fruit juice has been recognized as a novel food in the European Union [65]. Evidence showed that Noni fruit juice had a preventive effect against cerebral ischemic neuronal damage in a mice model [66]. Muto et al. [67] also reported that Noni juice protected mice brains from stress induced cognitive dysfunction, predominantly reducing the blood vessel density caused by stress. The administration of an ethyl acetate extract of noni fruit increased serotonin, dopamine, and antioxidant-enzyme serum levels in mice model with beta-amyloid induced cognitive dysfunction [68]. The ethanol extract of Noni fruit also improved memory, brain blood flow, and attenuated oxidative stress, acetylcholinesterase activity in a mice model [69]. A behavioral test revealed that the administration of the methanolic extract of Noni fruits decreased the negative effects of heroin and alcohol dependence [70, 71]. Despite a number of experimental evidence related to nervous system disorders, no specific natural product from this species has so far been identified and evaluated against nervous system disorders.

*Withania somnifera*, "Ashwagandha", is an undershrub commonly used in the traditional medicine of Bangladesh, naturally occurring in the North Bengal region. Among the 23 species of genus *Withania*, Ashwagandha is the most highly valued medicinal plant in traditional medicine and has been used since more than 3000 years. Various uses of this species including nervous system disorders (tonic, senile debility, nervous tension, loss of memory) reflect the ethno-pharmacological importance. Recent studies also demonstrated its multiple activities on nervous system disorders, particularly neuritic regeneration activity [72], neuroprotective activity [73], anti-anxiety and anti-depression activity [74], anti-Parkinson’s activity [75], nootropic and anti-Alzheimer’s activity [76], and anti-convulsant effects [77]. Roots are the most frequently used parts and the compounds isolated from these roots are effective against nervous system disorders. For example, withanolide A and withanoside IV (steroidal lactones) (Fig. 10) attenuated the β-amyloid (25–35) protein with the hope of enabling Alzheimer’s disease management [78, 79]. In an in vivo experimental report, it has been demonstrated that bioactive glyco-withanolides (Fig. 10) enhanced the activity levels of various antioxidant enzymes in the frontal cortex and striatum of rats, which may also be relevant for Alzheimer’s disease therapy [80].

## Plants Used Against CNS Disorder: Economical and Botanical Context

Apart from the medicinal benefits, many of the mentioned species are economically important and cultivated or collected as part of Bangladeshi tradition. Many medicinal plant species have also other uses such as foodstuff, in cosmetics and hygiene, as additives in different preparations, as part of rituals, and as medicines for ailments not related to the CNS.

The fruits of many medicinal plant species, including *Aegle marmelos*, *Citrullus lanatus*, *Citrus grandis*, *Phoenix sylvestris*, *Phyllanthus embelica*, *Solanum torvum*, and *Terminalia chebula*, are predominantly used as foods. The same holds true for various green leaves commonly consumed as vegetables namely, *Alpinia nigra*, *Amaranthus viridis*, *Bacopa monnieri*, *Centella asiatica*, *Coccinia grandis*, *Ipomoea aquatica*, *Moringa oleifera*, and *Nelumbo nucifera*. *Aloe vera*, *Curcuma longa*, *Curcuma aromatica*, and *Santalum album* are natural cosmetics used in Bangladesh since centuries. Spices are substances with pungent and aromatic properties used to flavor foods or beverages. *Cissus repens*, *Curcuma longa*, *Dillenia indica*, *Kaempferia galanga*, *Ocimum americanum*, and *Ocimum gratissimum* are common spices used in different curries and beverages. Species used as ornament (*Tabennaemontana divaricata*), masticatory substances (*Achyranthes aspera, Areca catechu*, and *Piper betel*), aquatic plants (*Nelumbo nucifera*), and incense plants (*Santalum album*) are sometimes included in the management of nervous system disorder [37].

## Future Prospects

Traditional plant-derived medicines are used throughout the world for a range of nervous disorders and may offer leads for drug development. In the past, native people around the world have helped to introduce many plant-derived products currently used to treat nervous disorders. Galanthamine (Fig. 4), a drug used against Alzheimer’s disease, is a natural alkaloid and was first isolated from *Galanthus nivalis*. Evidence-based and safe use of non-expensive plant-derived medications against nervous disorders may offer an enormous public health benefit, particularly for low-income countries. Research showed that fruit juice of noni (*Morinda citrifolia*, a traditional medicine of Bangladesh) has more inhibitory effects on hydrocephalus-induced degenerative disorders than memantine, a synthetic drug used against Alzheimer’s disease [81]. However, most of the pharmacological investigations carried out on the properties of the above-mentioned plants are only on a preliminary level. In addition, plant natural product as well as pharmacological potentials of many species mentioned in this review have not been scientifically examined at all yet.

It is therefore of pronounced interest to perform in-depth phyto-pharmacological assessments of traditionally used species to reveal potential new applications. This will additionally lead to a better understanding of traditional knowledge and clinical observations. For example, acteoside (Fig. 12) previously isolated from *Clerodendrum infortunatum* [82] and recently has been proved as an efficacious natural product against neurocytotoxicity, cognitive deficit, and neurochemical disturbances [83]. On the other hand, semisynthetic modifications of old and new natural compounds may yield substances for therapy, which are more effective than the genuine natural products they are derived from. One notable example is rivastigmine, which is more active than physostigmine (Fig. 4) (originally isolated from *Physostigma venenosum* Balf.) in the treatment of Alzheimer's and Parkinson's disease. Moreover, the multifactorial nature of Alzheimer’s disease suggests that a multi-targeted therapeutic approach might be more advantageous than single target drugs and combination therapies. This review shows that *Bacopa monnieri, Citrus grandis, Piper betel,* and *Withania somnifera* have an interesting activity against different biomarkers of Alzheimer’s disease and have distinct mechanism of action (Table 2). A combined therapy of these species or their bioactive natural products may contribute to an all-encompassing treatment strategy for Alzheimer’s disease. At the same time, combinatory herbal therapy could be more beneficial for those who are suffering from multiple nervous disorders.

## Conclusion

In many fields, traditional medicinal knowledge offers interesting leads for pharmacological research. Bangladesh is abundant in medicinal plants with various ethno-medicinal uses. In this review, we have compiled data on a large number of plant species, used as traditional medicine against neurological problems in Bangladesh. Many of these species have also displayed activity in bioassays matching their traditional uses. Based on these observations, future extensive investigations on those particular species can be targeted to identify the compounds responsible for the observed bioactivities as well as to unravel their mechanisms of action. Up to date, only a few of those active natural products and their respective modes of action have been identified (Table 2). We hope that the findings compiled in this review will contribute to the successful usage of ethno-medicinal knowledge of medicinal plants and their bioactive natural products in the treatment of CNS disorders.

## Abbreviations

CAM: Complementary and alternative medicine
CNS: Central nervous system
FDA: Food and drug administration
TBM: Traditional Bangladeshi medicine
T&CM: Traditional and complementary medicine
WHO: World Health Organization
